# Population-Level Transitions in Observed Difficulties Through Childhood and Adolescence

**DOI:** 10.1037/dev0001874

**Published:** 2025-02-27

**Authors:** Elia Benhamou, Danyal Akarca, Joe Bathelt, Sue Fletcher-Watson, Duncan E. Astle

**Affiliations:** 1 MRC Cognition and Brain Sciences Unit, University of Cambridge; 2 Department of Psychology, Royal Holloway, University of London; 3 Department of Psychology, University of Amsterdam; 4 The Salvesen Mindroom Research Centre, University of Edinburgh; 5 Department of Psychiatry, University of Cambridge

**Keywords:** development, machine learning, Millennium Cohort study, childhood, adolescence

## Abstract

In an attempt to better characterize the complexity of difficulties observed within developing populations, numerous data-driven techniques have been applied to large mixed data sets. However, many have failed to incorporate the core role of developmental time in these approaches, that is, the typical course of change in behavioral features that occurs over childhood to adolescence. In this study, we utilized manifold projections alongside a gradient-boosting model on data collected from the Millennium Cohort Study to unpack the central role of developmental time in how behavioral difficulties transition between the ages of 5, 11, and 17. Our analysis highlights numerous observations: (a) Girls develop relatively greater internalized behavioral problems during adolescence; (b) in the case of a chaotic home environment, co-occurring internalizing and externalizing difficulties tend to persist during childhood; (c) peer problems were the most likely to persist over the whole 12-year period (especially in the presence of early maternal depression and poor family relationships); and (d) there were two pathways with distinct risk factors leading to antisocial behaviors in adolescence—an early-childhood onset pathway and later adolescent onset pathway. Our findings provide evidence that investigations of child and adolescent difficulties must be open to the possibility of multiple subgroups and variability in trajectory over time. We further highlight the crucial role of family and social support and school experience-related factors in predicting children’s outcomes.

## Public Significance Statement

Using a large sample of data from the Millennium Cohort Study, followed from the age of 9 months to 17 years, we use data-driven methods to examine developmental change in children’s observed behaviors as they grow up. We identify clusters of children over more than a decade and establish key factors that predict longitudinally particular profiles and transitions from childhood into adolescence.

Existing classification systems that seek to categorize neurodevelopmental, psychiatric, and mental health conditions into discrete diagnostic entities have been widely critiqued (Astle et al., 2022; Cuthbert & Insel, 2013; Dalgleish et al., 2020; Etkin & Cuthbert, 2014). These systems struggle to accommodate the variability within, and shared features across, categories. They also struggle to accommodate profiles that do not neatly map onto diagnostic criteria but which are nonetheless impactful. As a result, people may be prevented to access support because their pattern of difficulties are not easily classified within existing structures (Hallett & Crompton, 2018). In an attempt to recharacterize the range and complexity of difficulties observed and experienced within a population, data-driven techniques such as latent class, factor analyses, or hierarchical clustering have been applied to large mixed data sets, which include clinical measures but also cognitive, sociodemographic, home–life, and mental health measurements. Cognitive or behavioral dimensions identified with latent class (Lacey et al., 2022; Swift et al., 2016), analyses of covariance (Dolan & Lennox, 2013), or exploratory factor analysis (Furlong et al., 2018; Holmes et al., 2021) can capture broad axes of variation and thereby provide a parsimonious account of the sample. In addition, clustering methods are increasingly being used to identify subgroups with similar characteristics, which may cut across established diagnostic categories (Archibald et al., 2013; Astle et al., 2019; Kushki et al., 2019).

However, these approaches—diverse samples combined with data-driven analysis—have largely ignored the role of *developmental time*. Where time has been considered, variability between individuals, between measures, and across time are each considered separately (Jacob et al., 2019; Wardenaar & de Jonge, 2013). This is problematic because behavioral problems seem to follow distinct developmental courses (Lewis & Rudolph, 2014). For instance, most children aged 3–5 with conduct and hyperactivity difficulties do not experience these difficulties by age 9–11 (Moffitt, 2006). Likewise, the prevalence of anxiety symptoms is high in late childhood, decreases in early adolescence, and then increases again from middle adolescence onward (Copeland et al., 2014; Van Oort et al., 2009). In short, behavioral difficulties need to be understood within their distinctive developmental context.

Studies that have examined developmental change have tended to focus on a single behavioral characteristic of interest. However, co-occurrence rates are high within neurodevelopmental and child psychiatric populations (Caspi & Moffitt, 2018; Hansen et al., 2018). For example, prevalence data for children with an attention-deficit/hyperactivity disorder (ADHD) diagnosis reveal that 40%–75% also meet criteria for oppositional defiant disorder, 14%–24% for conduct disorder, and approximately one third for an anxiety disorder (Mayes et al., 2009). To complicate matters further, the co-occurrence of problems fluctuates over time (Franke et al., 2018). In ADHD, new co-occurring difficulties tend to accumulate during adolescence and adulthood, for example, anxiety disorders, obsessive–compulsive disorder, and conduct disorders (Taurines et al., 2010). In contrast, some difficulties that often co-occur with the original presentation dissipate, for example, ADHD often co-occurs with a tic disorder, but the presentation of the tics usually improves substantially over developmental time (Black et al., 2021).

In one example, Bathelt et al. (2021) used a data-driven approach to capture the range, complexity, and co-occurrence of behavioral difficulties at two points in development. Using a population-level birth cohort, they followed 6,744 individuals as they transitioned from childhood to adolescence. They first identified latent dimensions representing parental ratings of behavioral difficulties and then used these to hierarchically cluster children at each time point. This allowed them to chart developmental changes in the frequency, presentation, and co-occurrence of behavioral difficulties. More importantly, it enabled the mapping of longitudinal transitions between clusters and the identification of environmental factors predicting particular transitions.

The purpose of the present study was to follow the behavioral profiles of children longitudinally from the ages of 5–17. Our aim was to chart not only changes in the overall range and complexity of difficulties but to map longitudinal transitions in profile. We used the Millennium Cohort Study (MCS), a nationally representative U.K. birth cohort of approximately 19,000 individuals, and focused on three pivotal developmental points: early childhood (age 5), late childhood (age 11), and adolescence (age 17). Behavioral difficulties likely change in nature between these ages, with known pronounced changes in physical features (e.g., puberty), brain structure, function (e.g., maturation of frontal lobe with gray matter volumes peaking at 11 years old followed by synaptic pruning and myelination; Blakemore & Choudhury, 2006), cognitive abilities (e.g., language development, problem solving, attention span, risk taking; Best & Miller, 2010; Schoon et al., 2010), social context (e.g., group identity, peer pressure; Blakemore, 2012), and environmental context (e.g., parenting, family responsibility, school setting; Furman & Buhrmester, 1992).

We focused our analysis on the validated derived subscales of the parent-reported Strength and Difficulties (SDQ) questionnaire, that is, conduct, hyperactivity, peer, emotional, and antisocial problems. After reducing the dimensional space using a uniform manifold approximation and projection (UMAP), we used *k*-means clustering to then identify subgroups of children and adolescents with different behavioral profiles within this reduced space. We then characterized the most common cluster transitions from early to late childhood and from late childhood to adolescence. Finally, using a regularized form of gradient-boosting algorithm for classification (XGBoost), we subsequently identified longitudinal and cross-sectional risk factors of these transitions using the preceding data sets from when the children were 9 months old and 3, 5, 7, and 11 years old.

## Method

### Participants

Data were taken from the MCS, a longitudinal cohort study of young people in the United Kingdom born between September 2000 and January 2002. MCS participants were randomly selected from U.K. child benefit records, and sampling was stratified to ensure adequate representation of the four U.K. countries, ethnic minorities, and highly deprived areas. Data from cohort members (CMs) and their families, including health, mental health, behavioral, socioemotional, education, and employment have been collected since participants were 9 months old. Seven sweeps have been collected at the time of writing, with another sweep planned for 2022. The present study draws on data from three sweeps: Sweep 3 (5 years old; *n* = 14,059; 50% female), Sweep 5 (11 years old; *n* = 12,751; 50% female), and Sweep 7 (17 years old; *n* = 9,191; 50% female; see Table 1 for demographic characteristics of each sweep). We also used data from the 9-month sweep (Sweep 1) and the 3-year-old sweep (Sweep 2) to assess risk factors for behavioral transitions related to circumstances and outcomes of birth and early childhood. All participants included in our analysis had complete responses to the SDQ. We excluded cases with missing information about age and gender. In our risk factor analysis, there were slight variations in the sample size due to small differences in missingness when combining all preceding and concurrent sweeps together. Actual sample sizes will be reported alongside the results.[Table tbl1]

### Measures

Table 2 lists all tests administered at each sweep.[Table tbl2]

#### SDQ Behavioral Profiles

Behavioral profiles were obtained from Goodman’s SDQ (Goodman, 1997) completed by the main caregiver at ages 5, 11, and 17 years old. The SDQ contains five subscales: Emotional Symptoms, Conduct Problems, Hyperactivity, Peer Problems, and Prosocial Behaviors (reversed to Antisocial Sehavior in this study). Example items from each subscale include: “Often unhappy, down-hearted or tearful” (Emotional Symptoms); “Often has temper tantrums or hot tempers” (Conduct Problems); “Restless, overactive, cannot stay still for long” (Hyperactivity); “Generally liked by other children” (Peer Problems); and “Shares readily with other children (treats, toys, pencils etc.)” (Prosocial Behaviors). Scores ranging from 0 to 10 for each of the five subscales are obtained by adding up parental responses from each of its five items, which are rated on a 3-point Likert-type scale (0 = *not true*, 1 = *somewhat true*, or 2 = *certainly true*). After reverse scoring the original Prosocial subscale, higher scores reflect greater difficulties. The validated three-band categorization was used to define two groups of children: (a) presenting with difficulties in any of the five SDQ subscales (part of the “borderline” or “abnormal” bandings), referred to as the “elevated” group, having relatively high difficulty scores, and (b) those who did not have any parent-reported difficulties across any scale, referred to as the “non-elevated” group.

Numerous studies have demonstrated the convergent and discriminant validity of the SDQ (van Widenfelt et al., 2003; Vogels et al., 2011). Specifically, when compared to the gold standard Child Behavior Checklist or Youth Self-Report scale(s), the SDQ scales are more strongly associated with its conceptually similar Child Behavior Checklist/Youth Self-Report scale(s) than with conceptually different Child Behavior Checklist/Youth Self-Report scales (for more details, see Vugteveen et al., 2021). The SDQ has also been validated across multiple clinical groups. For instance, for ADHD, the Hyperactivity/Inattention scale has a sensitivity of 91% and a specificity of 90%, and for autism, three scales combine to provide a sensitivity of 79% and specificity of 93% (Russell et al., 2013). However, it is important to recognize that it is not valid for *all* conditions. In a community sample of almost 8,000 5- to 15-year-olds, the SDQ identified individuals with a psychiatric diagnosis with a specificity of 95% and a sensitivity of 63%. However, the questionnaire was markedly better at detecting individuals with conduct, hyperactivity, depressive, and some anxiety disorders, identifying over 70% of them. But this detection rate drops to below 50% for those with specific phobias, separation anxiety, and eating disorders (Goodman et al., 2000).

#### Cognitive Performance

A detailed guide on the cognitive measures used within the MCS study is available (Moulton et al., 2020). Specifically, direct assessment of cognitive performance and mental health were obtained at the three time points. The earlier sweeps of the MCS consisted predominantly of the British Ability Scale II (BAS II) measures (Elliott et al., 1996).

The cognitive assessment for MCS3 (Sweep 3; 5 years old) consisted of:

1 BAS II, Naming Vocabulary. This is a verbal test which assessed the spoken vocabulary of the child. Test items consist of colored pictures of objects shown one at a time, and the cohort member responds verbally as to what the object is. The test comprises of 36 items (pictures of objectives) in total and has been used to predict delayed language development from 3 years (Law et al., 2012).
2 BAS II, Pattern Construction. This is a nonverbal, spatial, problem-solving test. For each item, a pattern was presented to the cohort member, and the cohort member was asked to replicate the pattern using flat form squats or solid plastic cubes with black and yellow patterns on each side. The test comprises of 23 items and has been used in modeling early-years school attainment (Sullivan et al., 2013).
3 BAS II, Picture Similarity. This is a nonverbal pictorial reasoning test. For each item, the cohort member was shown a row of four pictures or designs, and the cohort member placed a fifth card below the stimulus picture it best matched. The test comprises of 33 items and has been investigated in its moderating role on family and neighborhood risk (Flouri et al., 2015).

The cognitive assessment for MCS5 (Sweep 5; 11 years old) consisted of:

1 BAS II, Verbal Similarities (Elliott et al., 1996). This is a verbal test that assessed the child’s verbal reasoning using verbal concepts. The child was given three stimulus words and asked to name the class to which all the examples belong. The test comprises of 35 items.
2 CANTAB Cambridge Gambling Task (Atkinson, 2015). This assessed the child’s decision-making and risk-taking behavior. The child would be asked to bet on the number of points to bet on before the decision is made. Intelligence has been associated with risk adjustment and quality of decision making derived from this task (Flouri et al., 2019).

The cognitive assessment for MCS7 (Sweep 7; 17 years old) consisted of:

1 Granada Learning Assessment Number Analogies (Granada Learning Assessment, 2024). This assessed the young person’s arithmetic knowledge and reasoning with numbers.

#### Mental Health

Mental health metrics were also different across age sweeps. At age 5, we used two subscales of the Child Social Behavior Questionnaire completed by the main caregiver measuring the child’s independence and self-regulating behavior and the child’s emotional dysregulation (Hartman et al., 2006). Items included in these subscales include “Likes to work things out for self” and “Easily frustrated,” respectively.

At ages 11 and 17, a shortened five-item Rosenberg Self-Esteem scale was also asked of cohort members (Rosenberg, 1965). Eleven-year-old children’s answer to the question “How do you feel about your life as a whole?” was used as a proxy for overall happiness level (1 = *completely happy* to 7 = *not happy at all*).

A range of different mental health measures were added at age 17, including the self-completed Neuroticism subscale of the Big Five personality traits (often listed under the acronym OCEAN: Openness, Conscientiousness, Extraversion, Agreeableness, and Neuroticism), assessing the young person’s emotional reactiveness and vulnerability to stress; the self-completed six-item Kessler scale, which measures non-specific psychological distress (Kessler et al., 2003), including items such as “During the last 30 days, about how often did you feel so depressed that nothing could cheer you up?”; the self-completed, short, seven-item Young Person Warwick–Edinburgh Mental Well-being Scale (Stewart-Brown et al., 2009) which provides a single summary score indicating overall well-being, including items such as “I’ve been feeling optimistic about the future”; the answer to whether cohort members have ever been diagnosed with depression and/or serious anxiety; and the answer to whether they have ever attempted to end their life.

#### Longitudinal and Cross-Sectional Risk Analysis

Having used the measures above to compare groups after clustering, our second analysis drew on a large number of factors, looking at longitudinal and cross-sectional risk factors for behavioral transitions between the ages of 5, 11, and 17 years old. We selected these on the basis of the extant literature. We included factors from all MCS sweeps, including MCS1 (Sweep 1; 9 months old) and MCS2 (Sweep 2; 3 years old), preceding or concurrent to the transition “source” sweep. In other words, these factors could originate from any time up and until the “take off” point for the behavioral transition.

Factors were grouped into nine domains: peri- and postnatal factors (birth weight, type of delivery, feeding problems; Bhutta et al., 2002; McCormick et al., 1990; Serati et al., 2017; Wiles et al., 2006), household factors (e.g., housing tenure, neighborhood safety, average family income; Adjei et al., 2022; Clair, 2019, p. 20; Miller et al., 2021; Minh et al., 2017), child physical health (e.g., BMI, sleep; Griffiths et al., 2011; Heron et al., 2013; Holley et al., 2011; Turnbull et al., 2013), child well-being (e.g., reluctance to go to school, self-esteem; Donnellan et al., 2005; Egger et al., 2003), child cognition (e.g., language development, mathematical abilities; Baird et al., 2022; Quistberg & Mueller, 2020; Thompson et al., 2019), caregiver health and mental health (e.g., Kessler scale, use of recreational drugs, alcohol consumption; Adjei et al., 2022; Maselko et al., 2015; Meadows et al., 2007; Schepman et al., 2011), caregiver relations (e.g., marital status, satisfaction with partner; Giannakopoulos et al., 2009), child relations (e.g., time spent outside with friends, time spent on social media; Brunborg & Burdzovic Andreas, 2019; Van Roy et al., 2010), and caregiver–child relationship (e.g., style of parenting, closeness to child; Bloomfield & Kendall, 2012; Coldwell et al., 2006). See Supplemental Table 3 for the full list of factors.

### Analysis Procedure

**Figure 1 fig1:**
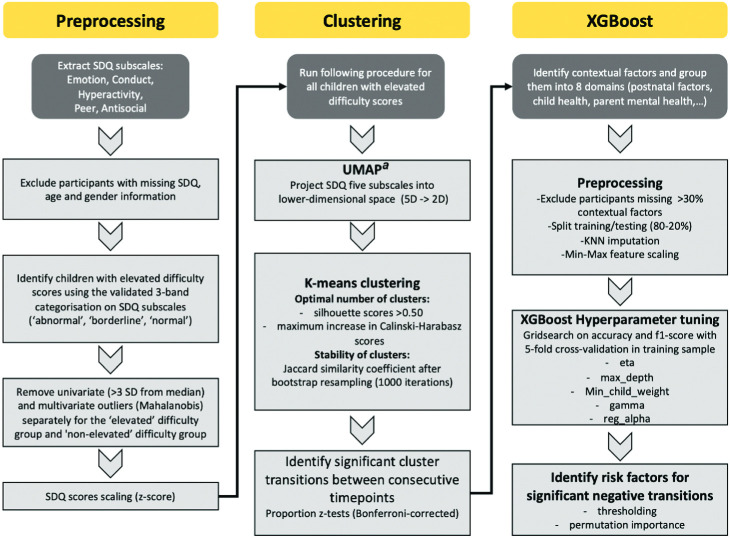
Flow Diagram Summarizing the Analysis Pipeline *Note*. XGBoost = gradient-boosting algorithm for classification; SDQ = Strength and Difficulties Questionnaire; UMAP = uniform manifold approximation and projection; KNN = *k*-nearest neighbor; max_depth = maximum depth (of our tree); min_child_weight = minimum child weight (minimum sum of instance weight needed for a child of our tree); reg_alpha = regularisation alpha (L1 regularisation term on weights). See the online article for the color version of this figure. ^a^ In the UMAP projection of SDQ, data from five dimensions (5D) to two dimensions (2D) reduce the size of the data but in a way that we do not lose out an essential information underlying the data. Subsequent figures present the 2D plot of each subject’s reduced 2D data.

Our analysis pipeline was designed first to clean the data and partition it into those with elevated and non-elevated difficulty scores on the SDQ (note that elevated here includes children with borderline or elevated scores on at least one scale). Then, with the elevated group, we conducted a dimension reduction approach called UMAP, before clustering, identifying significant transitions across development, and then establishing significant risk factors for these transitions (Figure 1; Table 3). Readers may wonder why we partitioned the data to focus on those with elevated scores. This is necessary because otherwise the dimensional space is dominated by individuals with scores within the normal range and not optimized to capture behavioral difficulties. In turn, the data present lower dimensionality because it is dominated by the extreme differences in overall severity across the whole sample and is then very difficult to cluster meaningfully.[Fig fig1][Table tbl3]

#### Clustering

The first step of our analysis was to identify subgroups of children within the elevated sample at each time point by using a clustering method on *z*-scored SDQ derived values. Before clustering, we first excluded any participants with missing SDQ data or missing age or gender information. We then partitioned our children into the elevated and non-elevated difficulty groups. We then removed univariate (>3 *SD*s from the median) and multivariate outliers (Mahalanobis distance >α quantile of the chi-square distribution with 5 *df*) separately for each group. The total number of children excluded at each time point is indicated in the consort diagrams in Supplemental Figures 1–3 (MCS 3: n = 702 or 4.7%, MCS 5: n = 60 or 0.5%, MCS 7: n = 61 or 0.7%, for univariate or multivariate outliers or because of missing information).

To avoid clustering in a sparse dimensional space and to improve clustering performance (Dalmaijer et al., 2021), we then projected the five SDQ subscales into a lower dimensional space using UMAP. This method is based on Riemannian geometry and algebraic topology and has been show to outperform Principle Component Analysis, t-distributed Stochastic Neighbor Embedding, or Multidimensional Scaling methods for (a) its ability to retain higher order, nonlinear interaction of variables inherent in the type of data we are studying; (b) its preservation of both local and global structure of the original data; (c) its greater flexibility with several distance metrics to choose from; (d) its computational efficiency (O[N] vs. O[Nlog(N)]); and (e) more reproducible results (Yang et al., 2021, p. 20).

Briefly, UMAP first constructs a topological representation of the original data with local manifold approximations, and then it optimizes the lower dimension embedding by minimizing the cross-entropy between the new lower dimensional space and the original higher dimensional space. We set UMAP parameters with a relatively high number of neighbors (*n*_neighbors = 50) to decrease the probability of producing fine-grained cluster structure that is more prone to be a result of patterns of noise. We set a low minimum distance (min_dist = 0.01) to make denser clusters and cleaner separations. We also used a correlation distance metric. UMAP was implemented using the UMAP V.0.3.2 Python implementation (McInnes et al., 2020).

We then used *k*-means clustering on the UMAP data in the lower dimensional space using the scikit-learn implementation in Python. Optimal number of clusters were chosen based on silhouette coefficients exceeding 0.5 and a steep increase in the Calinski–Harabasz index, both indicating a good separation between clusters scores (Bathelt et al., 2021; Dalmaijer et al., 2021), and Jaccard similarity coefficients between clusters obtained after bootstrap resampling (1,000 iterations) and the original clusters (Zumel & Mount, 2014) exceeding 0.85, indicating a good stability of clusters.

The non-elevated portion of the data was included as a comparison group for each age point. Comparisons between clusters for gender, cognition, mental health, and neurodivergence diagnosis were conducted with chi-square tests and one-way analysis of variance with Bonferroni post hoc *t* tests. All clusters (including the comparison non-elevated group) were compared to the whole sample at each age point.

#### Assessment of Transitions

Proportion *z* tests (Bonferroni-corrected for multiple comparisons) were used to compare the proportion of participants transitioning from one cluster at one time point to another cluster at the next time point with an equal-split transition (e.g., in the case of five clusters, an equal split would be 20% of the sample transitioning into each cluster). Only significant transitions above the equal-split proportion, that is, transitions that occurred more than we would expect by chance, will be reported here. To make sure transitions were not driven by higher Euclidean distance from the center of each identified cluster, we compared the distribution of silhouette scores for each participant who was part of a significant transition to the distribution of silhouette scores for the entire cluster (see Supplemental Figures 4 and 5). In other words, borderline cases could switch between clusters over time not because of any meaningful change in behavior but because of imprecision in the clustering solution. Comparing the silhouette scores in this way controls for this.

#### Identification of Longitudinal Risk Factors for Significant Transitions

Longitudinal and concurrent predictors of significant behavioral transitions between the ages of 5–11 years old and between the ages of 11–17 years old were identified looking at the feature importance gain after performing XGBoost on our train samples (80% of our data set) using the XGBoost Python package. For each significant transition, we compared the subgroup of children with a particularly elevated difficulty profile transitioning to another elevated difficulty profile cluster at the following time point (“transition of interest”) to the subgroup of children with the same elevated difficulty profile transitioning to the non-elevated difficulty scores group at the following time point (“control transition”). In essence, we were looking to identify factors that distinguish those who transition to a different profile of behavioral difficulty from those who transition to no apparent difficulties, given the same starting profile. XGBoost is an ensemble algorithm based on gradient-boosted trees. It integrates the predictions of “weak” classifiers to achieve a “strong” classifier (tree model) via a serial training process. It incorporates an L1 (Least Absolute Shrinkage and Selection Operator) regression regularization, which prevents the model from overfitting and is robust to multicollinearity of features.

We first removed all participants missing more than 30% of the features (Uh et al., 2021; 282 and 178 participants were excluded for the “5–11” cohort and the “11–17” cohort, respectively), and after splitting our data set into training and testing sets (80%–20% as we prioritized low variance of our parameter estimates rather than our performance statistics), we imputed missing data using *k*-nearest neighbor imputation and scaled features using Min–Max method (3.35% and 3.30% of data were imputed for the 5–11 cohort and the 11–17 cohort, respectively). We then tuned the following hyperparameters optimizing both accuracy and *f*1-score using a GridSearch procedure with 5-fold cross validation on our training sample: nrounds (the number of sequentially built trees in the model), η (the learning rate), max_depth (the maximum levels deep that each tree can be grown), min_child_weight (the minimum degree of impurity needed in a node before a split is attempted), γ (the minimum amount of splitting by which a node must improve the predictions), and reg_alpha (the L1 regularization term). We also set the scale_pos_weight hyperparameter with the inverse of the class distribution to tune the behavior of the algorithm for imbalanced classification problems. We then identified the optimal feature importance score threshold and number of features, which led to the best model performance accuracy and *f*1-score for predicting the transition of interest in our test sample. Permutation importance was finally implemented on the test data by sequentially replacing each feature with its random shuffling and measuring at which extent permutation of a particular feature affected the accuracy of predictions. Only the features that survived both thresholding and permutation importance were considered genuine risk factors for a significant behavioral transition. We chose to report feature importance gain in this article, which corresponds to the improvement in accuracy brought by a feature to the branches it is on.

The data and code that support the findings of this study are available from the corresponding author upon reasonable request.

### Transparency and Openness

We report all data exclusions, all data transformations, and all measures in the study, and we follow the Journal Article Reporting Standards (Appelbaum et al., 2018). All analysis code is available at https://github.com/eliacello/MCS_BehaviouralTransitions_2021/tree/main. The code in this repository was used to clean, preprocess, cluster, and model data from the Millennium Cohort Study, known as “Child of the New Century,” and freely accessible through the UK Data Service (University College London, UCL Social Research Institute, Centre for Longitudinal Studies, 2024). The Millennium Cohort Study data are not included in the repository. Data were analyzed using R, Version 4.0.0 (R Core Team, 2020), and the packages EFA.dimensions Version 0.1.8.4 (https://CRAN.R-project.org/package=EFA.dimensions), ggplot Version 3.5.1 (Wickham, 2016), polycor Version 0.8-1 (https://CRAN.R-project.org/package=polycor), and ez Version 4.4-0 (https://CRAN.R-project.org/package=ez). Data were also analysed using Python, Version 3.10, and the libraries numpy, pandas, matplotlib, seabird, sklearn (preprocessing, metrics, cluster), scipy, umap, and statistics. This study’s design and its analysis were not preregistered.

## Results

**Figure 2 fig2:**
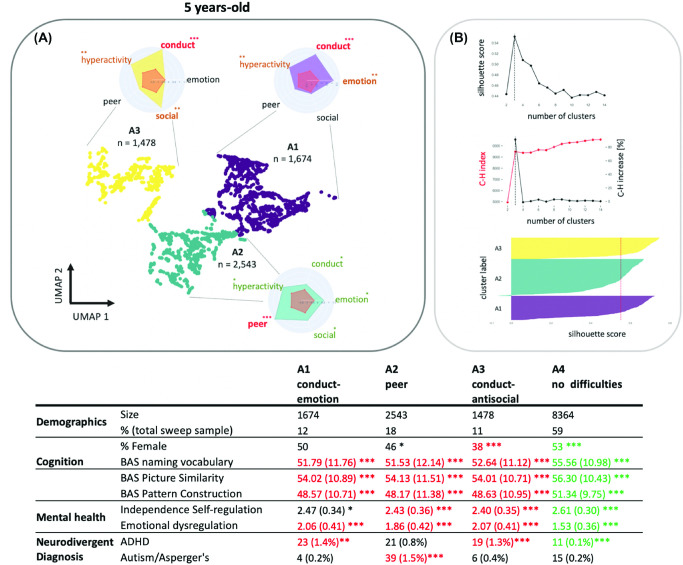
Results of the k-Means Clustering of the Early Childhood Data *Note*. (A) The reduced 2D UMAP space on which each participant Strength and Difficulties Questionnaire-derived variable were projected. Clusters are coded with colors with radar plots indicating the profiles of behavioral ratings for each cluster. (B) The average silhouette for different number of clusters (top), the Calinski–Harabasz index for different cluster numbers (middle), and the silhouette coefficient for each group (bottom). The table gives an overview of size, gender ratio, cognitive assessments, incidence of mental health problems, and neurodivergent diagnosis for each identified cluster. Green indicates the values above the whole sample average (or below for “negative” domains such as emotional dysregulation). Red indicates the values below the whole sample average (or above for “negative” domains such as emotional dysregulation). UMAP = uniform manifold approximation and projection; BAS = British Ability Scale; ADHD = attention-deficit/hyperactivity disorder. See the online article for the color version of this figure. The asterisks in (A) indicate Cohen’s d relative to μ = 0 and the standard deviation across all groups: * *d* > 0.5 (green). ** *d* > 1 (amber). *** *d* > 1.5 (red). Asterisks in the table indicate the statistical comparison to the whole Bonferroni-corrected sample: * *p* < .05. ** *p* < .01. *** *p* < .001.

**Figure 3 fig3:**
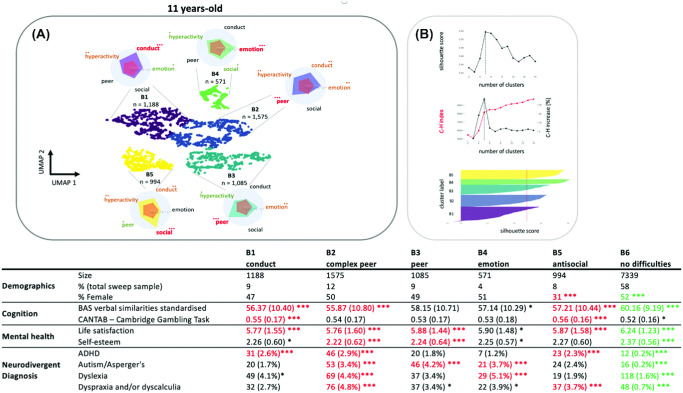
Results of the k-Means Clustering of the Late Childhood Data *Note*. (A) The reduced 2D UMAP space on which each participant Strength and Difficulties Questionnaire-derived variable were projected. Clusters are coded with colors with radar plots indicating the profiles of behavioral ratings for each cluster. (B) The average silhouette for different number of clusters (top), the Calinski–Harabasz index for different cluster numbers (middle), and the silhouette coefficient for each group (bottom). The table gives an overview of size, gender ratio, cognitive assessments, incidence of mental health problems, and neurodivergent diagnosis for each identified cluster. Green indicates the values above the whole sample average (or below for “negative” domains such as emotional dysregulation). Red indicates the values below the whole sample average (or above for “negative” domains such as emotional dysregulation). UMAP = uniform manifold approximation and projection; BAS = British Ability Scale; ADHD = attention-deficit/hyperactivity disorder. See the online article for the color version of this figure. The asterisks in (A) indicate Cohen’s d relative to μ = 0 and the standard deviation across all groups: * *d* > 0.5 (green). ** *d* > 1 (amber). *** *d* > 1.5 (red). Asterisks in the table indicate the statistical comparison to the whole Bonferroni-corrected sample: * *p* < .05. **p < .01. *** *p* < .001.

**Figure 4 fig4:**
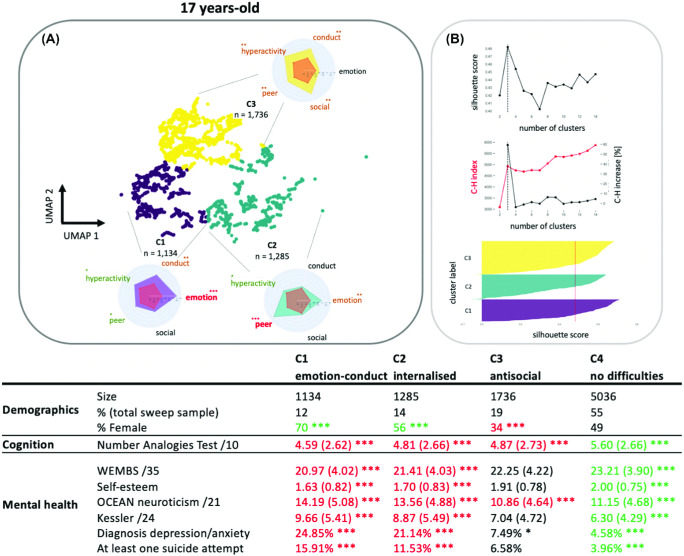
Results of the k-Means Clustering of the Adolescence Data *Note*. (A) The reduced 2D UMAP space on which each participant Strength and Difficulties Questionnaire-derived variable were projected. Clusters are coded with colors with radar plots indicating the profiles of behavioral ratings for each cluster. (B) The average silhouette for different number of clusters (top), the Calinski–Harabasz index for different cluster numbers (middle), and the silhouette coefficient for each group (bottom). The table gives an overview of size, gender ratio, cognitive assessments, incidence of mental health problems, and neurodivergent diagnosis for each identified cluster. Green indicates the values above the whole sample average (or below for “negative” domains such as emotional dysregulation). Red indicates the values below the whole sample average (or above for “negative” domains such as emotional dysregulation). UMAP = uniform manifold approximation and projection; BAS = British Ability Scale; ADHD = attention-deficit/hyperactivity disorder. See the online article for the color version of this figure. The asterisks in (A) indicate Cohen’s d relative to μ = 0 and the standard deviation across all groups: * *d* > 0.5 (green). ** *d* > 1 (amber). *** *d* > 1.5 (red). Asterisks in the table indicate the statistical comparison to the whole Bonferroni-corrected sample: * *p* < .05. ** *p* < .01. *** *p* < .001.

Our dimensionality reduction and clustering analysis identified three clusters at age 5, five clusters at age 11, and three clusters at age 17. For these cluster solutions, silhouette coefficients all exceeded 0.5, we observed a steep increase in the Calinski–Harabasz index, and the Jaccard similarity coefficients all exceeded 0.85 after bootstrapping. See Figures 2–4 for an illustration of the behavioral profiles and descriptive statistics for each age group, respectively.[Fig fig2][Fig fig3][Fig fig4]

### Cluster Comparisons

#### Early Childhood

For the early childhood sweep (age 5), the first cluster (A1, *n* = 1,674) scored highly on both the Conduct Problems and Emotional Problems subscales; the second cluster (A2, *n* = 2,543) was principally defined by high antisocial behavior scores alone, and the third cluster (A3, *n* = 1,478) scored highly for conduct problems and antisocial behaviors. There were more girls in the non-elevated group (identified before clustering; A4, *n* = 8,364) and more boys in A2 and A3 compared to the whole sample gender distribution. The clusters showed differences in cognition and emotion regulation abilities, one-way analysis of variance: BAS naming: *F*(3, 14055) = 121.8, *p* < .001; BAS similarities: *F*(3, 14028) = 48.98, *p* < .001; BAS pattern: *F*(3, 14029) = 92.02, *p* < .001; Children’s Social Behavior Questionnaire (CSBQ) independence: *F*(3, 14055) = 356.3, *p* < .001; CSBQ emotional dysregulation: *F*(3, 14054) = 1,652, *p* < .001). Post hoc tests revealed that all groups significantly differed from the whole sample (*p* < .05, Bonferroni corrected): Children with non-elevated difficulty scores had higher cognitive and CSBQ scores than the whole sample whereas children with behavioral problems had significantly reduced cognitive and CSBQ scores. The prevalence of neurodevelopmental diagnoses was also different across groups with significantly more ADHD children diagnosed in clusters A1 and A3 presenting with conduct problems, fewer children diagnosed with ADHD in A4 presenting with non-elevated difficulty scores (A1: χ^2^ = 16.2, *p* < .001; A2: χ^2^ = 11.7, *p* < .001; A4: χ^2^ = 20.6, *p* < .001), and more children diagnosed with autism in cluster A2 (χ^2^ = 38.8, *p* < .001) presenting with peer relationship difficulties.

#### Late Childhood

For the late childhood sweep (age 11), the first cluster (B1, *n* = 1,330) scored highly for conduct problems mainly; the second cluster (B2, *n* = 1,463) was defined by high scores on a whole range of subscales mainly involving peer relationships problems, and at a lesser extent, hyperactivity, conduct, and emotional problems; the third cluster (B3, *n* = 1,085) scored highly for internalized problems mainly weighted toward peer problems (and less toward emotional dysregulation); the fourth cluster (B4, *n* = 539) had high emotional problems scores; and the last cluster (B5, *n* = 996) scored highly for antisocial behaviors mainly. There were more girls in the non-elevated group without behavioral problems (B6, *n* = 7,339) and more boys in the group with antisocial behavior compared to the whole sample gender distribution. The clusters also showed differences in cognition, verbal similarities: *F*(5, 12491) = 78.53, *p* < .001; Cambridge Gambling Task risk taking: *F*(5, 12050) = 13.5, *p* < .001, and Bonferroni-corrected post hoc *t* tests indicated that children with conduct problems (cluster B1) scored lower at the verbal task and were more prone for risk-taking behaviors compared to the whole sample (*p* < .001). Children with complex peer problems (cluster B2) had also significantly reduced verbal scores (*p* < .05). Mental health also differed between groups, satisfaction with life: *F*(5, 12328) = 53.08, *p* < .001; self-esteem: *F*(5, 12123) = 30.26, *p* < .001, with all groups with behavioral problems (clusters B1–B4), except the one displaying antisocial behaviors, showing reduced overall satisfaction with life and self-esteem; children with non-elevated difficulty scores (B6) increased satisfaction with life and self-esteem compared to the whole sample (*p*_Bonferroni_ < 0.05). Finally, the proportion of children diagnosed with neurodevelopmental disorders differed between clusters (ADHD: χ^2^ = 20.6, *p* < .001; autism: χ^2^ = 20.6, *p* < .001; dyslexia: χ^2^ = 20.6, *p* < .001; dyspraxia: χ^2^ = 20.6, *p* < .001).

#### Adolescence

For the adolescence sweep (age 17), the first cluster (C1, *n* = 1,134) scored highly on the Emotional subscale and, to a lesser extent, on the Conduct subscale; the second cluster (C2, *n* = 1,285) indicated high scores for peer problems; and the third cluster (C3, *n* = 1,736) scored highly for antisocial problems and, to a lesser extent, peer relationship, hyperactivity, and conduct problems. There were more girls in the group with internalizing problems (C1 and C2) and more boys in the group with complex antisocial behavior (C3) compared to the whole sample gender distribution. The clusters showed differences in arithmetic abilities, *F*(3, 8423) = 68.27, *p*_Bonferroni_ < 0.001, and Bonferroni-corrected post hoc *t* tests indicated that adolescents with non-elevated difficulty scores (C4) had higher cognitive scores than the whole sample whereas children with behavioral problems had significantly reduced cognitive scores (*p* < .001).The groups showed overall difference for several measures of mental health, Warwick–Edinburgh Mental Wellbeing Scale: *F*(3, 8645) = 134.4, *p* < .001; Kessler scale: *F*(3, 8686) = 209.6, *p* < .001; Openness, Conscientiousness, Extraversion, Agreeableness, and Neuroticism scale: *F*(3, 8687) = 195.6, *p* < .001; self-esteem: *F*(3, 8681) = 94.33, *p* < .001; diagnosis of depression and/or anxiety: χ^*2*^ = 635.2, *p* < .001; proportion of adolescents having attempted suicide: χ^2^ = 250.66, *p* < .001. As for the preceding sweep, clusters with behavioral problems (clusters C1 and C2), except the one displaying complex antisocial behaviors, showed lower mental health on all scales while children in the non-elevated group (C4) showed higher mental health compared to the whole sample (*p*_Bonferroni_ < 0.05).

### Transitions

**Figure 5 fig5:**
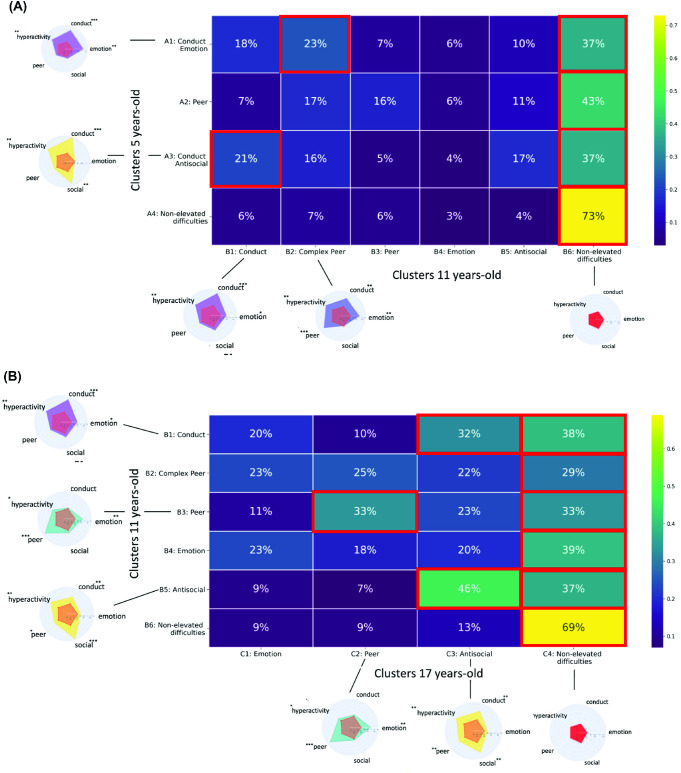
Overview of Transitions Showing the Percentage of Children Transitioning Between Groups *Note*. These groups were identified in (A) early childhood (5 years old; rows) to groups identified in late childhood (11 years old; columns) and from groups identified in (B) late childhood (rows) to groups in adolescence (17 years old; columns). Cells with red rectangles indicate proportions that were significantly above what was expected by chance (Bonferroni-corrected). See the online article for the color version of this figure.

The validity of our clusters was also reflected in the patterns seen in the behavioral transitions across childhood and adolescence. Figure 5 describes the proportion of participants transitioning from one cluster to another across time. For all elevated clusters found at ages 5 and 11, a significant proportion transitioned to the non-elevated group. At age 5, a larger-than-expected proportion of children (23%) transitioned from having conduct and emotional problems (cluster A1) to having problems forming peer relationships co-occurring with hyperactivity, conduct, and emotional problems (cluster B2) at age 11. A significant proportion of children aged 5 (21%) also transitioned from having conduct problems combined with antisocial behaviors (cluster A3) to having conduct problems alone (cluster B1).[Fig fig5]

At age 11, a larger-than-expected 46% of children displaying antisocial behaviors (cluster B4) and 32% of children with conduct problems (cluster B1) transitioned to a group of adolescents having antisocial problems co-occurring with peer relationship, hyperactivity, and conduct problems (cluster C3). A larger-than-expected 33% of children having difficulties forming peer relationships transitioned to a group of adolescents with the same peer relationship problems co-occurring with emotional problems (cluster C2).

The comparison of silhouette scores distribution between children from a “significant” transition (e.g., 23% going from A1 to B2) and the rest of the cluster (e.g., A1) can be found in the Supplemental Figures 4 and 5.

### Longitudinal and Cross-Sectional Risk Factors

We next applied the classification model XGBoost to determine the predictors for the significant behavioral transitions listed in the previous section, contrasting transition of interest (elevated difficulty cluster to elevated difficulty cluster) to its corresponding control transition (elevated difficulty cluster to non-elevated cluster). The strength of features surviving thresholding and permutation in cross-validated data sets corresponds to the importance gain (*g*), which corresponds to the improvement in accuracy brought by a feature to the branches of the decision tree it is on.

**Figure 6 fig6:**
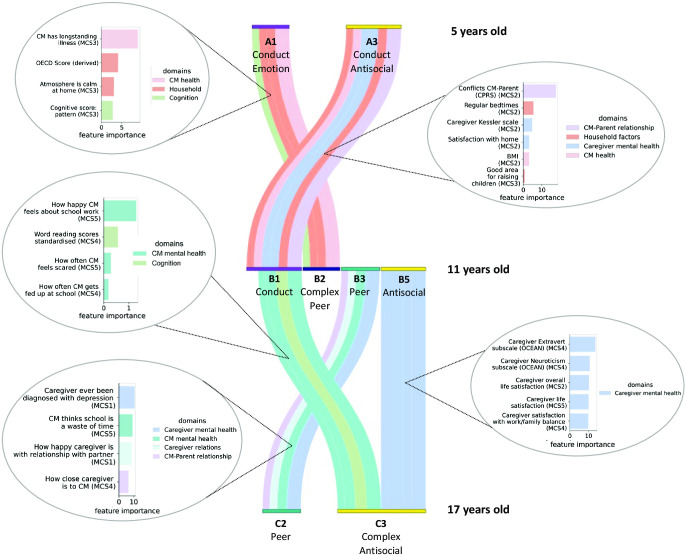
Sankey Diagram Showing Risk Factors That Predict Significant Transitions *Note*. Colors in both Sankey diagram and bar plots correspond to risk factors domains, and the thickness of color lines between clusters indicates the importance gain associated to the risk factor. Transitions to the nonelevated difficulty scores group are omitted from the figure. MCS = Millennium Cohort Study; CPRS = Child-Parent Relationship Scale; CM = cohort member; OECD = Organisation for Economic Co-Operation and Development. See the online article for the color version of this figure.

We summarize our findings in Table 4 and Figure 6. At age 5, the strongest predictors for transitioning from having conduct and emotional problems (cluster A1) to having problems forming peer relationships co-occurring with hyperactivity, conduct, and emotional problems (cluster B2) at age 11 were having long-standing illness (*g* = 9.00), a low Organisation for Economic Co-operation and Development family income (*g* = 4.11), an agitated atmosphere at home (*g* = 3.08), and a lower cognitive score for pattern construction (*g* = 2.78). Conversely, the persistence of conduct problems between 5 and 11 years old was best predicted by a set of factors measured at age 3: having caregivers reporting a difficult relationship with their child (*g* = 17.64), irregular bedtimes (*g* = 6.30), a high BMI (*g* = 6.60), caregiver’s high psychological distress (*g* = 7.78), caregiver’s low satisfaction with home (*g* = 5.61), and caregiver considering the neighborhood a disadvantaged area to raise kids (*g* = 4.20). At age 11, the persistence of antisocial behaviors with the addition of new difficulties with peer relationships, hyperactivity, and conduct problems was best predicted by caregiver’s mental health measured when the child was 3, 7, and 11 years old: caregivers self-reporting lower extraversion (*g* = 13.61), a higher emotional reactiveness and vulnerability to stress (*g* = 10.68), a lower overall life satisfaction (*g* = 10.25; *g* = 9.98), and a lower satisfaction with work/family balance (*g* = 9.87). The transition from having conduct problems at age 11 to having a combination of conduct, hyperactivity, peer relationship difficulties, and antisocial behaviors at age 17 was best predicted by the child’s mental health mainly linked with school at ages 7 and 11 and reading abilities at age 7: self-reported feeling unhappy about school work (*g* = 10.61), often getting fed up at school (*g* = 6.54), often feeling scared (*g* = 8.54), and lower age-standardized word reading scores (*g* = 9.19). Finally, the persistence of internalized problems (difficulties with peer relationships and emotional dysregulation) across adolescence was best predicted by the main caregiver’s diagnosis of depression at least once in their lifetime (*g* = 1.29), the main caregiver feeling unhappy with their relationship with partner (g = 0.28; reported when the child was 9 months old), the caregiver reporting not being close to their child at age 7 (*g* = 0.19), and the child considering school a waste of time (*g* = 0.56) at age 11.[Table tbl4][Fig fig6]

## Discussion

Behavioral difficulties change radically in their range, scope, and complexity across development, something poorly captured by rigid diagnostic frameworks. By integrating a dimension reduction and clustering paradigm with a subset of the population-representative MCS, we found distinct subgroups of children based on parental reports of emotional, conduct, hyperactivity, peer, and social difficulties. The number of clusters and their respective profiles varied across developmental time. We identified several common cluster-to-cluster transitions between early childhood and late childhood and between late childhood and adolescence. Most children transitioned to the non-elevated difficulty scores group, but a significant minority of children were at elevated risk of persistent difficulties or of developing new difficulties over time. Our subsequent analysis of longitudinal risk revealed that a diverse range of socioeconomic, health problems, poor caregiver mental health, and challenging household factors predicted transitions during childhood. Poor mental health of both caregiver and adolescents accompanied with difficulty in child–parent and parent–parent relationships explained the most common behavioral transitions during adolescence.

### Gender Gap

There is a shift in gender distribution across our clusters between childhood and adolescence: While there were systematically more girls in the portion of the sample with non-elevated behavioral problems at ages 5 and 11, there was an equal gender representation by 17 years old. Furthermore, there was an overrepresentation of girls in clusters with internalized problems at age 17 (70% female in the group with emotional problems and 56% female in the group with peer problems). Conversely, there were systematically more boys in the groups displaying conduct and antisocial behaviors throughout childhood and adolescence. In other words, the proportion of females being members of the “elevated scores” portion of our data set increases with age, driven mostly by increasing internalizing difficulties. Given the reliance on parent-reported data in this study and the fact that internalized difficulties are by their nature less detectable by others, it is unclear whether the difficulties themselves increase in this time period, or parents become more aware of them.

The gradual shift in gender distribution replicates the well-known “gender gap” in behavioral problems during adolescence: Girls in late adolescence are at least twice as likely as boys to become depressed (Wade et al., 2002) or anxious (Zahn-Waxler et al., 2000) while boys generally have greater odds of developing externalizing problems than internalizing problems (Kovess-Masfety et al., 2016, 2021).

In addition to highlighting the gendered differences in the prevalence and presentation of parent-rated behavioral difficulties, our data-driven approach also confirms the particular importance of adolescence as a crucial transition for girls. This confirms a finding also present in other large-scale U.K.-based population-level studies that instead rely on a diagnostic framework (e.g., Ford et al., 2003). But *why* does adolescence present such a crucial developmental stage for the emergence of internalizing disorders, most especially in girls and young women? We can conceive of two broad categories of (nonmutually exclusive) possible explanations. First, there are substantial social changes that happen around this time, which may change the nature of interpersonal relationships and social structures. The most obvious example of this is school transitions that commonly occur around early and midadolescence in the United Kingdom. Contemporaneous data that look at the role of school-level factors show that characteristics of that secondary school environment (e.g., location, “school climate”) are predictive of well-being (Ford et al., 2021), something observed in other countries also (Nielsen et al., 2017). Second, there could be significant biological changes, such as hormonal changes, that play a role in driving the increase in the prevalence of internalizing difficulties particularly in young women (see Patton & Viner, 2007, for a review). Our study is not in a position to disentangle these potential influences, but it is nonetheless important to note that the gendered pattern and developmental timing that emerges from our novel data-driven approach aligns well with other large-scale studies covering this age range.

### Co-Occurrence of Externalizing and Internalizing Problems in Early Childhood Predicts Complex Peer Problems in Late Childhood

In our sample, almost a quarter of children with co-occurring externalizing (conduct) and internalizing (emotional problems) problems at age 5 continued displaying a constellation of both externalizing and internalizing problems at 11 years old. Co-occurrence of externalizing and internalizing symptoms is most often found in early childhood (Fanti & Henrich, 2010; Lilienfeld, 2003; Oland & Shaw, 2005). However, little is known about their long-term prospective associations with developmental outcomes, as the literature predominantly focuses on internalizing or externalizing problems only and not their interaction over time. A few studies have examined the consequences of co-occurrence of externalizing and internalizing problems on general functioning in emerging adulthood (Arslan et al., 2021; Sourander et al., 2007). However, no studies have carefully followed up children capturing such broad areas of difficulty and assessing their trajectories in later stages of childhood or adolescence.

While most children entering preschool have developed a set of strategies to cope with their emotions and manage their behavior in demanding situations, our study suggests that children displaying broad behavioral problems at this age might find it harder to establish good coping strategies, which in turn could result in additional co-occurring difficulties over time, with a high toll on peer relationships. It has been shown that difficulties attaining adaptive strategies for emotional self-regulation during preschool age have various consequences such as notably diminished perceived social competence, lower peer status, and more externalizing problems (Blandon et al., 2010; Denham et al., 2003; Kim & Cicchetti, 2010). However, most studies look at behavioral problems in isolation while our findings suggest a link between the breadth of difficulties co-occurring in preschool age and the experience of isolation and rejection in peer contexts 6 years later.

Across this same period, from 5 to 11 years old, we found that the persistence of undifferentiated problems during childhood is related to a diverse range of socioeconomic and child health factors. The common denominator to these factors was a relatively high environmental unpredictability: a chaotic home environment, low income, and child’s chronic illness—which usually requires lifestyle changes and continuous behavioral adaptation to the unpredictable course of the illness. Lower executive function was also a significant predictor of the persistence of problems over childhood, but this could be a direct consequence of the child’s exposure to an unpredictable environment as repeatedly shown in previous studies (Campbell et al., 2009; Compas et al., 2012; Ferguson et al., 2013; LeBlanc et al., 2003; Skinner & Zimmer-Gembeck, 2007). Investigating the links between neurocognitive delays associated with childhood illness should be a high priority as altered trajectories of brain development may delay the deployment of the effective coping skills necessary to alleviate the occurrence of emotional and behavioral problems. But it is important to note that it is particularly difficult to establish causality, despite the temporal sequencing of predictors and their associated transitions. There could be crucial shared genetic predictors between children and parents, which themselves may significantly covary with (Trzaskowski et al., 2014) or interact with (Price & Jaffee, 2008) early environmental stressors.

### Two Pathways Leading to Complex Antisocial Behaviors in Late Adolescence

We identified two pathways leading to antisocial behavior at 17 years old: The first pathway tracked by children who started developing antisocial behaviors for the first time at age 11 while the second pathway comprised children with persistent conduct problems since 5 years of age. At first glance, this mirrors Moffit’s developmental taxonomy of antisocial behavior, which posits an “adolescent-limited” pathway and a “life course persistent” pathway (Moffitt, 1993). This is an important demonstration of variability of trajectory because the approach to studying antisocial behavior has hitherto largely assumed a single grouping. For example, our understanding of the role of relationship dynamics, parenting practices, cultural and community context (Dishion & Patterson, 2006), but also environmental and genetic factors (Gard et al., 2019; Wesseldijk et al., 2018), are based upon the assumption of a single etiological pathway. To our knowledge, the present study is the first to assess the risk factors for more than one possible trajectory. However, our findings do not fully align with Moffit’s developmental taxonomy, according to which young people on the adolescent-limited pathway engage in antisocial behaviors as a rebellion against adult “privilege” as they become aware of those privileges but are yet unable to access them (Moffitt et al., 1996). By contrast, in our findings, this trajectory was best predicted by a caregiver’s poor mental health and not by any socioeconomic factors or mental or physical health of adolescents themselves. One possibility is that this is because a parent’s own mental health shapes their ability to rate, or perception of, their offspring’s antisocial behavior or vice versa. Rather than situating the cause of adolescent antisocial behavior in the individual, more attention should be paid to the challenges they face in their relationships and environment.

Our childhood-onset antisocial group at 17 years old displayed lower cognitive scores in all domains and higher risk-taking scores on the gambling task (Tieskens et al., 2018). The risk factors predicting the persistence of antisocial behaviors during adolescence were related to general anxiety and antischool feelings as well as specific difficulties in reading. This latter predictor replicates several studies showing how language and reading difficulties increase the risk for behavioral problems notably by affecting social competence and moral development (Ketelaars et al., 2010; Piotrowska et al., 2019; Yew & O’Kearney, 2013). The antischool risk factor for antisocial behavior is also in accordance with recent studies showing the importance of a positive school climate for promoting a young person’s sense of community, cooperation, supportive ties among students, and more generally, spontaneous prosocial behavior (Thapa et al., 2013). It has notably been shown that adolescents reporting higher levels of perceived positive school climate at age 12 showed higher levels of prosocial behaviors over time (Luengo Kanacri et al., 2017). From this, it might be tempting to conclude that interventions should focus on improving that school environment and the young person’s perception of it. However, given that this group also experiences educational difficulties that can themselves be isolating, often incurring social exclusion and stigma (e.g., Haft et al., 2023), it is likely that effective interventions also need to consider a more multisystemic approach that also supports academic development across both school and home settings.

Finally, membership of the “childhood-onset” antisocial pathway at age 11 was related to a more diverse range of risk factors, including parent–child conflicts, inconsistent discipline, high child’s BMI score, poor caregiver’s mental health, and caregivers’ negative view of the neighborhood. These results closely match the childhood risk factors predicting future antisocial behavior found in another U.K. population-representative cohort (Avon Longitudinal Study of Parents and Children), that is, maternal hostility toward the child, maternal depression, mothers’ negative view of their neighborhood, and difficulty paying the rent (Stevens, 2018). In our case, all factors were identified when the child was only 3 years old, which confirms the need for early family support interventions to prevent long-lasting antisocial behaviors that are likely to persist during adulthood and develop into antisocial personality disorders.

### Persistence of Peer Problems Over Childhood and Adolescence

Peer problems were more likely to persist over childhood and adolescence than other behavioral difficulties we measured. At age 11, children had either peer problems in isolation or in combination with high scores on subscales measuring both internalized and externalized problems (emotional, conduct, and hyperactivity). Both groups principally originated from the subgroup with peer problems at age 5, meaning that some children maintain peer problems over time while others accumulate evidence of a broader range of difficulties. A significant proportion of children with isolated peer problems at age 11 then transitioned to a group with the exact same problems at age 17, which was predicted by a combination of poor caregiver’s mental health and a difficult relationship with both partner and child, preceding the transition by 10 years (when the child was only 9 months old) and 4 years. Perinatal depression has repetitively been linked to poorer child development outcomes, and our study confirms the need for maternal depression interventions to prevent the persistence of peer problems in their child and adolescent (Maselko et al., 2015). Programs targeting parenting are also the leading early intervention strategy for the prevention of child behavior problems. These are typically rooted in social learning theory, which suggests that children’s behavior is shaped by the behavior they observe in their caregivers (Bandura & Walters, 1963; Ryan et al., 2017). In our study, it is possible that both caregivers’ relationship and child–parent relationship (closeness) act as maladaptive models for the early adolescent to initiate and foster healthy relationships with their peers. However, it is also possible, because of our reliance on parent-reported data to capture child difficulties, that parents with poor mental health and a difficult relationship with their child systematically perceive their child more negatively than parents and caregivers with better mental health and relationships. Another possibility is that shared genetic factors between children and parents may increase the risk of developing psychosocial difficulties in the presence of an unstable early environment.

### Limitations

There are several limitations to this study. Although SDQ subscales have been repeatedly validated, they combine separate constructs in the same scale, such as inattention and impulsivity in the Hyperactivity scale or anxiety and depressive states in the Emotional Problems scale. Identifying dimensions based on more granular questionnaire items could capture more variability between individuals presenting with graduated levels of functioning and could potentially bring us closer to revealing underlying mechanisms.

Differential attrition between sweeps of data collection may have introduced a bias as children with fewer behavioral problems were more likely to provide data in adolescence. Consequently, the prevalence of behavioral difficulties in adolescence might be underestimated in the present study. Furthermore, this analysis was based on SDQ parent ratings as these were the only ratings consistently available across time points. Self-reported or teacher ratings might help disambiguate some of our results, especially where child behavior is related to parent mental health and parent–child relationships. Similar potential for bias applies when reporting mental health problems, which were provided either by parents or adolescents themselves, using a different set of questionnaires at each time point. Direct assessments from specialist professionals, structured interviews, and a systematic use of the same questionnaires at all time points will be needed to get a more accurate account of mental health problems incidence in childhood and adolescence. Moreover, as a U.K.-representative cohort, our sample was over 90% White: examining the impact of ethnicity on these trajectories in the United Kingdom would require oversampling of non-White participants. Although ethnicity was included as a potential risk factor in our transition analysis, we still need to replicate our study with international samples to investigate potential interactions between ethnicity and incidence of behavioral difficulties. Finally, soft clustering algorithms or latent variable models may provide more accurate allocation of participants to clusters incorporating probabilistic assignment instead of hard categorization. Although this approach might be more representative of reality, it could also dramatically increase the complexity of the analysis.

### Conclusion

Establishing how children and young people’s patterns of difficulties shift over time, and what predicts those shifts, is a crucial step to identify individuals in need of support and to move from a reactive to a preventive model of mental health and well-being. Using data-driven clustering and a regularizing gradient-boosting model, we identified key transitions between the age of 5–17 years old. We showed that there was an increasing number of girls developing internalized behavioral problems during adolescence, that co-occurring internalizing and externalizing difficulties persisted during childhood in the case of a chaotic home environment, that peer problems were most likely to persist over this 12 year-period (especially in the presence of early maternal depression and poor family relationships), and finally that there were two pathways with distinct risk factors leading to antisocial behaviors in adolescence—childhood onset and adolescent onset. These findings provide evidence that investigations of child and adolescent difficulties must be open to the possibility of multiple subgroups and variability in trajectory over time. They also highlight the crucial role of family and social support and school experience factors in predicting children’s outcomes, rather than situating the cause of observed difficulties solely within the young person themselves.

## Supplementary Material

10.1037/dev0001874.supp

## Figures and Tables

**Table 1 tbl1:** Demographic Characteristics of MCS3 (5 Years Old), MCS5 (11 Years Old), and MCS7 (17 Years Old) Samples

Characteristic	Sweep 3 (age 5)	Sweep 5 (age 11)	Sweep 7 (age 17)
*n* (included)	14,059	12,751	9,191
Age, years (*SD*)	5.22 (0.25)	10.67 (0.48)	17.17 (0.33)
Female (%)	7,053 (50)	6,329 (50)	4,605 (50)
Country of birth (%)			
England	8,856 (63)	8,248 (65)	6,164 (67)
Scotland	1,728 (12)	1,442 (11)	957 (10)
Wales	2035 (15)	1,786 (14)	1,249 (14)
Northern Ireland	1,440 (10)	1,274 (10)	821 (9)
Ethnicity (%)			
White	12,072 (86)	10,882 (85)	5,593 (83)
Mixed	397 (3)	110 (0.9)	50 (0.7)
Pakistani and Bangladeshi	669 (5)	820 (6)	310 (5)
Indian	335 (2)	301 (2)	162 (2)
Black African, Caribbean or other	421 (3)	408 (3)	174 (3)
Other Asian	94 (0.7)	123 (1)	72 (1)
Other ethnic group	66 (0.5)	89 (0.7)	44 (0.6)
OECD below 60% median poverty (%)	4,452 (32)	3,040 (24)	NaN
*Note.* Refer to Supplemental Tables 1 and 2 for analysis of attrition between sweeps. MCS = Millennium Cohort Study; MCS3 = MCS (Sweep 3); MCS5 = MCS5 (Sweep 5); MCS7 = MCS7 (Sweep 7); OECD = Organisation for Economic Co-operation and Development; NaN = not a number.			

**Table 2 tbl2:** List of Tests Administered at Each Sweep

Behavior	Sweep 3 (age 5) SDQ	Sweep 5 (age 11) SDQ	Sweep 7 (age 17) SDQ
Cognition	BAS Naming vocabulary/BAS pattern construction and picture similarity	BAS verbal similarities/CANTAB Cambridge Gambling Task	Number Analogies Test—Cognitive Abilities Test 3
Mental health	Child Social Behavior Questionnaire (independence and self-regulating behavior/emotional dysregulation)	Five-item Rosenberg self-esteem scale/“How do you feel about your life as a whole?”	Five-item Rosenberg self-esteem scale/OCEAN (Neuroticism subscale)/six-item Kessler scale/seven-item young person Warwick–Edinburgh Mental Wellbeing Scale/“Have you ever been diagnosed with depression and/or serious anxiety?”/“Have you ever attempted to end your life?”
*Note*. SDQ = Strength and Difficulties Questionnaire; BAS = British Ability Scale; OCEAN = Openness, Conscientiousness, Extraversion, Agreeableness, and Neuroticism.			

**Table 3 tbl3:** Summarized Information of Key Steps Within the Analysis Pipeline

Step	Further detail
1. Clean and partition SDQ data	Create two groups of participants, identifying those with elevated parent-reported difficulties. Ensure a complete and high-quality data set for subsequent analysis.
2. UMAP	Reduce the dimensional space for subsequent clustering to maximize clustering effectiveness while retaining data complexity.
3. *k*-means clustering	Identify data-driven subgroups within the elevated sample, at each time point independently.
4. Proportion *z* tests	Identify the numbers of children who move between cluster over time, indicating a shift in their specific profile of difficulties with age. Identify significant behavioral transitions (higher proportion than predicted by chance).
5. Identify and clean contextual factors	Identify longitudinal and cross-sectional risk factors for behavioral transitions based on the extant literature and group them into domains. Ensure a high-quality data set.
6. XGBoost	Use a model that allows the use of multicollinear variables. Allow generalizability of model and avoid overfitting. Identify longitudinal and concurrent predictors of significant behavioral transitions.
*Note*. SDQ = Strength and Difficulties Questionnaire; UMAP = uniform manifold approximation and projection; XGBoost = Gradient-boosting algorithm for classification.	

**Table 4 tbl4:** Summary of Longitudinal and Cross-Sectional Risk Factors Linking to Transitions Over Time

Age transition	Transition of interest (%)	Control transition (%)	Predictor (age of child when reported; importance gain)
5–11	Conduct–emotion (A1) to complex peer (B2; 23%)	Conduct–emotion (A1) to nonelevated (B6; 37%)	Cohort member has longstanding illness (5; *g* = 9.00) Low Organisation for Economic Co-operation and Development family income (5; *g* = 4.11) Agitated atmosphere at home (5; *g* = 3.08) Lower cognitive score for pattern construction (5; *g* = 2.78)
	Conduct–antisocial (A2) to conduct (B1; 21%)	Conduct–antisocial (A2) to nonelevated (B6; 37%)	Difficulty caregiver–child relationship (3; *g* = 17.64) Irregular bedtimes (3; *g* = 6.30) High child BMI (3; *g* = 6.60) High Caregiver Kessler (K6) score (3; *g* = 7.78) Caregiver’s low satisfaction with home (3; *g* = 5.61) Disadvantaged area to raise kids (5; *g* = 4.20)
11–17	Conduct (B1) to antisocial (C3; 32%)	Conduct (B1) to nonelevated (C4; 38%)	Feeling unhappy about school work (11; *g* = 10.61) Lower age-standardized word reading scores (7; *g* = 9.19) Often feeling scared (11; *g* = 8,54) Often getting fed up at school (7; *g* = 6.54)
	Peer (B3) to peer (C2; 33%)	Peer (B3) to nonelevated (C4; 33%)	Caregiver ever been diagnosed with depression (9 months; *g* = 1.29) Caregiver feeling unhappy with relationship with partner (9 months; *g* = 0.28) Caregiver not close to their child (7; *g* = 0.19) Child considering school a waste of time (11; *g* = 0.56)
	Antisocial (B5) to antisocial (C3; 46%)	Antisocial (B5) to nonelevated (C4; 37%)	Lower caregiver extraversion score (7; *g* = 13.61) High caregiver Neuroticism score (7; *g* = 10.68) Lower caregiver overall life satisfaction (3, 11; *g* = 10.25, *g* = 9.98) Lower caregiver work–family balance (7; *g* = 9.87)
