# Supplementary material for: Population-Level Transitions in Observed Difficulties Through Childhood and Adolescence
Source: Dev Psychol. 2025 Feb 27;61(8):1495–515. doi: 10.1037/dev0001874 (PMC12243395; doi:10.1037/dev0001874)
Supplement: Supplementary file 1 [file DEV-2023-0824_Supplemental_Materials.docx]

**SUPPLEMENTARY MATERIAL FOR:**

**Population-level transitions in observed difficulties through childhood and adolescence**

**Supplementary Figure 1**

*CONSORT diagram of data exclusion for the early childhood data (MCS3).*

**Supplementary Figure 2**

*CONSORT diagram of data exclusion for the late childhood data (MCS5).*

**Supplementary Figure 3**

*CONSORT diagram of data exclusion for the adolescence data (MCS7).*

**Supplementary Table 1**

*Demographic characteristics and comparison of MCS3 (5 year-olds) included in MCS5 (11 year-olds) participants with MCS3 participants absent from MCS5.*

|  | **Sweep 3 children included in Sweep 5** | **Sweep 3 children absent from Sweep 5** | **Statistical difference** |  |
| --- | --- | --- | --- | --- |
| **n (included)** | 11,173 | 2886 | NA |  |
| **Age [years] (SD)** | 5.21 (0.24) | 5.23 (0.26) | n.s. |  |
| **Female (%)** | 5620 (50) | 1328 (46) | n.s. |  |
| **Country of birth (%)** |  |  |  |  |
| England | 7098 (63) | 1758 (61) | n.s. |  |
| Scotland | 1325 (12) | 403 (14) | n.s. |  |
| Wales | 1605 (14) | 430 (15) | n.s. |  |
| Northern Ireland | 1145 (10) | 295 (10) | n.s. |  |
| **Ethnicity (%)** |  |  |  |  |
| White | 9732 (87) | 2340 (81) | n.s. |  |
| Mixed | 301 (3) | 96 (0.3) | n.s. |  |
| Pakistani and Bangladeshi | 498 (4) | 171 (6) | n.s. |  |
| Indian | 245 (2) | 90 (3) | n.s. |  |
| Black African, Caribbean or other | 283 (3) | 142 (5) | n.s. |  |
| Other Asian | 84 (0.7) | 23 (0.8) | n.s. |  |
| Other ethnic group | 43 (0.4) | 23 (0.8) | n.s. |  |
| **OECD below 60% median poverty (%)** | 3215 (29) | 1237 (24) | *** (X^2^ = 102.4, df = 1, p < 0.001) |  |
| **SDQ Emotion** | 1.27 (1.40) | 1.35 (1.44) | n.s. |  |
| **SDQ Conduct** | 1.42 (1.37) | 1.53 (1.45) | * (t = 3.84, df = 4038, p = 0.002) |  |
| **SDQ Hyperactivity** | 3.20 (2.27) | 3.49 (2.36) | *** (t = 6.02, df = 4084, p < 0.001) |  |
| **SDQ Peer** | 1.05 (1.23) | 1.19 (1.32) | *** (t = 5.20, df = 4017, p < 0.001) |  |
| **SDQ Social** | 1.53 (1.54) | 1.58 (1.59) | n.s. |  |

Statistical tests include Pearson's Chi-squared test and Welch Two Sample t-test with Bonferroni correction

N.B. 11-year-olds who were present in MCS5 but who missed the MCS3 timepoint were not included in this analysis as this analysis looks at attrition level

**Supplementary Table 2**

*Demographic characteristics and comparison of MCS3 (11 year-olds) included in MCS7 (17 year-olds) participants with MCS5 participants absent from MCS7.*

|  | **Sweep 5 children included in Sweep 7** | **Sweep 5 children absent from Sweep 7** | **Statistical difference** |  |
| --- | --- | --- | --- | --- |
| **n (included)** | 8479 | 4272 | NA |  |
| **Age [years] (SD)** | 10.66 (0.48) | 10.70 (0.47) | n.s. |  |
| **Female (%)** | 4293 (51) | 2036 (48) | n.s. |  |
| **Country of birth (%)** |  |  |  |  |
| England | 5640 (66) | 2608 (61) | n.s. |  |
| Scotland | 901 (11) | 541 (13) | n.s. |  |
| Wales | 1160 (14) | 626 (15) | n.s. |  |
| Northern Ireland | 778 (9) | 496 (12) | *** (X^2^ = 15.0, df = 1, p < 0.001) |  |
| **Ethnicity (%)** |  |  |  |  |
| White | 7221 (85) | 3661 (86) | n.s. |  |
| Mixed | 70 (0.8) | 40 (0.1) | n.s. |  |
| Pakistani and Bangladeshi | 560 (7) | 260 (6) | n.s. |  |
| Indian | 216 (2) | 85 (2) | n.s. |  |
| Black African, Caribbean or other | 248 (3) | 160 (4) | n.s. |  |
| Other Asian | 96 (1) | 27 (0.6) | n.s. |  |
| Other ethnic group | 56 (0.7) | 33 (0.8) | n.s. |  |
| **OECD below 60% median poverty (%)** | 1678 (20) | 1362 (24) | *** (X^2^ = 136.1, df = 1, p < 0.001) |  |
| **SDQ Emotion** | 1.79 (1.95) | 1.97 (2.01) | *** (t = 4.89, df = 8342, p < 0.001) |  |
| **SDQ Conduct** | 1.25 (1.43) | 1.56 (1.63) | *** (t = 10.57, df = 7642, p < 0.001) |  |
| **SDQ Hyperactivity** | 2.96 (2.40) | 3.39 (2.52) | *** (t = 9.28, df = 8200, p < 0.001) |  |
| **SDQ Peer** | 1.28 (1.60) | 1.48 (1.71) | *** (t = 6.42, df = 8085, p < 0.001) |  |
| **SDQ Social** | 1.16 (1.48) | 1.27 (1.61) | * (t = 3.83, df = 7945, p = 0.004) |  |

Statistical tests include Pearson's Chi-squared test and Welch Two Sample t-test with Bonferroni correction

N.B. 17-year-olds who were present in MCS7 but who missed the MCS5 timepoint were not included in this analysis as this analysis looks at attrition levels

**Supplementary Table 3**

*List of all variables used for XGBoost model to identify predictors of significant transitions. Cells colored in yellow indicate all factors found to predict significant transitions in childhood (5 to 11 years-old). Cells colored in amber indicate all factors found to predict significant transitions in adolescence (11 to 17 years-old).*

| **Sweep** | **Age** | **Factors** | **Domains** | **%Missing** |
| --- | --- | --- | --- | --- |
| MCS 1 | 9 months old | Whether caregiver has longstanding illness | Caregiver Health and Mental Health | 0.02% |
| MCS 1 | 9 months old | Whether caregiver has ever been diagnosed with depression | Caregiver Health and Mental Health | 0.02% |
| MCS 1 | 9 months old | Tired most of the time | Caregiver Health and Mental Health | 1.55% |
| MCS 1 | 9 months old | Often miserable or depressed | Caregiver Health and Mental Health | 1.53% |
| MCS 1 | 9 months old | Often worried about things | Caregiver Health and Mental Health | 1.52% |
| MCS 1 | 9 months old | Often gets in violent rage | Caregiver Health and Mental Health | 1.50% |
| MCS 1 | 9 months old | Suddenly scared for no good reason | Caregiver Health and Mental Health | 1.49% |
| MCS 1 | 9 months old | Easily upset or irritated | Caregiver Health and Mental Health | 1.62% |
| MCS 1 | 9 months old | Constantly keyed up or jittery | Caregiver Health and Mental Health | 1.61% |
| MCS 1 | 9 months old | Every little thing gets on nerve | Caregiver Health and Mental Health | 1.50% |
| MCS 1 | 9 months old | Heart often races | Caregiver Health and Mental Health | 1.50% |
| MCS 1 | 9 months old | Have no one to share feelings with | Caregiver Relations | 2.50% |
| MCS 1 | 9 months old | Partner is sensitive and aware | Caregiver Relations | 15.78% |
| MCS 1 | 9 months old | Partner does not listen | Caregiver Relations | 13.04% |
| MCS 1 | 9 months old | How often goes out as a couple | Caregiver Relations | 13.31% |
| MCS 1 | 9 months old | How happy are you with relationship | Caregiver Relations | 12.73% |
| MCS 1 | 9 months old | Partner ever used force | Caregiver Relations | 14.36% |
| MCS 1 | 9 months old | Housing tenure | Household | 0.64% |
| MCS 1 | 9 months old | Number of siblings in household | Household | 0.00% |
| MCS 1 | 9 months old | IMD 2004 Overall Decile | Household | 0.00% |
| MCS 1 | 9 months old | Income Domain Decile | Household | 0.00% |
| MCS 1 | 9 months old | Employment Domain Decile | Household | 0.00% |
| MCS 1 | 9 months old | Health Deprivation and Disability | Household | 0.00% |
| MCS 1 | 9 months old | Education Skills and Training | Household | 0.00% |
| MCS 1 | 9 months old | Barriers to Housing and Services | Household | 9.44% |
| MCS 1 | 9 months old | Living Environment Domain Decile | Household | 0.00% |
| MCS 1 | 9 months old | URI_Mixed Urban-Rural | Household | 0.00% |
| MCS 1 | 9 months old | URI_Rural | Household | 0.00% |
| MCS 1 | 9 months old | URI_Town and Fringe | Household | 0.00% |
| MCS 1 | 9 months old | URI_Urban | Household | 0.00% |
| MCS 1 | 9 months old | URI_Village, Hamlet & Isolated Dwellings | Household | 0.00% |
| MCS 1 | 9 months old | NS-SEC 5 classes | Household | 5.19% |
| MCS 1 | 9 months old | NVQ equivalent of highest academic qualification | Household | 9.42% |
| MCS 1 | 9 months old | Ethnicity_Black or Black British | Household | 0.00% |
| MCS 1 | 9 months old | Ethnicity_Indian | Household | 0.00% |
| MCS 1 | 9 months old | Ethnicity_Mixed | Household | 0.00% |
| MCS 1 | 9 months old | Ethnicity_Other Ethnic group (inc Chinese,Other) | Household | 0.00% |
| MCS 1 | 9 months old | Ethnicity_Pakistani and Bangladeshi | Household | 0.00% |
| MCS 1 | 9 months old | EAS_Non-working for other/unknown reason | Household | 0.00% |
| MCS 1 | 9 months old | EAS_Self employed | Household | 0.00% |
| MCS 1 | 9 months old | Gestation time | Peri and post-natal | 0.64% |
| MCS 1 | 9 months old | Weight at 9 months old | Peri and post-natal | 1.59% |
| MCS 1 | 9 months old | Parent's age at birth | Peri and post-natal | 0.00% |
| MCS 1 | 9 months old | Planned pregnancy | Peri and post-natal | 2.85% |
| MCS 1 | 9 months old | Duration of labour | Peri and post-natal | 7.06% |
| MCS 1 | 9 months old | Caregiver smokes | Peri and post-natal | 0.00% |
| MCS 1 | 9 months old | Whether lived apart from baby | Peri and post-natal | 0.03% |
| MCS 1 | 9 months old | Birth weight | Peri and post-natal | 13.75% |
| MCS 1 | 9 months old | Age (in weeks) when CM first had solid food | Peri and post-natal | 3.20% |
| MCS 1 | 9 months old | CM has all immunisations | Peri and post-natal | 3.82% |
| MCS 1 | 9 months old | Any problems with hearing | Peri and post-natal | 14.77% |
| MCS 1 | 9 months old | Number of health problems | Peri and post-natal | 2.75% |
| MCS 1 | 9 months old | Number of accidents or injuries | Peri and post-natal | 2.75% |
| MCS 1 | 9 months old | Number of hospital admissions | Peri and post-natal | 2.75% |
| MCS 1 | 9 months old | Frequency wakes up at night | Peri and post-natal | 2.76% |
| MCS 1 | 9 months old | Whether baby crying is a problem | Peri and post-natal | 4.08% |
| MCS 1 | 9 months old | Milk feeds at about the same time | Peri and post-natal | 5.65% |
| MCS 1 | 9 months old | Sleepy at about the same time each evening | Peri and post-natal | 4.45% |
| MCS 1 | 9 months old | Naps about the same length | Peri and post-natal | 5.07% |
| MCS 1 | 9 months old | Solid food at about the same time | Peri and post-natal | 4.60% |
| MCS 1 | 9 months old | Chest infections | Peri and post-natal | 0.00% |
| MCS 1 | 9 months old | Skin problems | Peri and post-natal | 0.00% |
| MCS 1 | 9 months old | Ear infections | Peri and post-natal | 0.00% |
| MCS 1 | 9 months old | Wheezing or asthma | Peri and post-natal | 0.00% |
| MCS 1 | 9 months old | Persistent or severe vomiting | Peri and post-natal | 0.00% |
| MCS 1 | 9 months old | Sight or eye problems | Peri and post-natal | 0.00% |
| MCS 1 | 9 months old | Feeding problems | Peri and post-natal | 0.00% |
| MCS 1 | 9 months old | Persistent or severe diarrhoea | Peri and post-natal | 0.00% |
| MCS 1 | 9 months old | Failure to gain weight or to grow | Peri and post-natal | 0.00% |
| MCS 1 | 9 months old | Fits or convulsions | Peri and post-natal | 0.00% |
| MCS 1 | 9 months old | Sleeping problems | Peri and post-natal | 0.00% |
| MCS 1 | 9 months old | Breathing difficulty or distress in first week | Peri and post-natal | 0.00% |
| MCS 1 | 9 months old | Delay in breathing at birth | Peri and post-natal | 0.00% |
| MCS 1 | 9 months old | Infection or suspected infection at birth | Peri and post-natal | 0.00% |
| MCS 1 | 9 months old | Jaundice requiring hospital treatment at birth | Peri and post-natal | 0.00% |
| MCS 1 | 9 months old | Other complications at birth | Peri and post-natal | 0.00% |
| MCS 1 | 9 months old | Breech birth - feet first | Peri and post-natal | 0.00% |
| MCS 1 | 9 months old | Foetal distress - Heart rate sign | Peri and post-natal | 0.00% |
| MCS 1 | 9 months old | Foetal distress - Meconium. other | Peri and post-natal | 0.00% |
| MCS 1 | 9 months old | Other complications during labour | Peri and post-natal | 0.00% |
| MCS 1 | 9 months old | Caesarian section | Peri and post-natal | 0.00% |
| MCS 1 | 9 months old | Cord around neck | Peri and post-natal | 0.00% |
| MCS 1 | 9 months old | Very long labour | Peri and post-natal | 0.00% |
| MCS 1 | 9 months old | Very rapid labour | Peri and post-natal | 0.00% |
| MCS 1 | 9 months old | Sleeps in a room with other children | Peri and post-natal | 0.00% |
| MCS 1 | 9 months old | Sleeps in parents bedroom | Peri and post-natal | 0.00% |
| MCS 2 | 3 years old | Caregiver general level of health (MCS2) | Caregiver Health and Mental Health | 0.02% |
| MCS 2 | 3 years old | Caregiver smokes (MCS2) | Caregiver Health and Mental Health | 0.02% |
| MCS 2 | 3 years old | Frequency caregiver consumes alcohol (MCS2) | Caregiver Health and Mental Health | 0.02% |
| MCS 2 | 3 years old | How often caregiver felt depressed in last 30 days (MCS2) | Caregiver Health and Mental Health | 9.80% |
| MCS 2 | 3 years old | Caregiver uses recreational drugs (MCS2) | Caregiver Health and Mental Health | 8.86% |
| MCS 2 | 3 years old | Overall life satisfaction (MCS2) | Caregiver Health and Mental Health | 8.45% |
| MCS 2 | 3 years old | Caregiver Kessler (K6) Scale (MCS2) | Caregiver Health and Mental Health | 8.12% |
| MCS 2 | 3 years old | Natural mother BMI (MCS2) | Caregiver Health and Mental Health | 8.68% |
| MCS 2 | 3 years old | How often caregiver spends time with friends (MCS2) | Caregiver Relations | 3.05% |
| MCS 2 | 3 years old | Caregiver has no one to share feelings with (MCS2) | Caregiver Relations | 11.47% |
| MCS 2 | 3 years old | Family would help in case of financial difficulties (MCS2) | Caregiver Relations | 12.17% |
| MCS 2 | 3 years old | Partner is sensitive and aware of caregiver needs (MCS2) | Caregiver Relations | 18.67% |
| MCS 2 | 3 years old | How often disagree over issues concerning CM (MCS2) | Caregiver Relations | 18.21% |
| MCS 2 | 3 years old | Partner ever used force in relationship (MCS2) | Caregiver Relations | 20.06% |
| MCS 2 | 3 years old | BMI (MCS2) | Child Health | 6.57% |
| MCS 2 | 3 years old | Overweight/Obseity Flag (MCS2) | Child Health | 6.57% |
| MCS 2 | 3 years old | Anyone smokes near CM (MCS2) | Child Health | 3.05% |
| MCS 2 | 3 years old | CM has longstanding illness (MCS2) | Child Health | 3.04% |
| MCS 2 | 3 years old | Concerns about CM speech/language (MCS2) | Child Health | 3.04% |
| MCS 2 | 3 years old | Parenting competence (MCS2) | CM-Parent Relationship | 8.86% |
| MCS 2 | 3 years old | Parenting: How often ignores CM when naughty (MCS2) | CM-Parent Relationship | 14.04% |
| MCS 2 | 3 years old | Parenting: How often smacks CM when naughty (MCS2) | CM-Parent Relationship | 11.85% |
| MCS 2 | 3 years old | Parenting: How often shouts at CM when naughty (MCS2) | CM-Parent Relationship | 12.23% |
| MCS 2 | 3 years old | Parenting: How often sends CM to bedroom when naughty (MCS2) | CM-Parent Relationship | 11.70% |
| MCS 2 | 3 years old | Parenting: How often takes away treats when naughty (MCS2) | CM-Parent Relationship | 12.85% |
| MCS 2 | 3 years old | Parenting: How often tells CM off when naughty (MCS2) | CM-Parent Relationship | 12.17% |
| MCS 2 | 3 years old | Parenting: How often bribes CM when naughty (MCS2) | CM-Parent Relationship | 11.82% |
| MCS 2 | 3 years old | Time spent with CM (MCS2) | CM-Parent Relationship | 0.27% |
| MCS 2 | 3 years old | Warm affectionate relationship with CM (MCS2) | CM-Parent Relationship | 6.89% |
| MCS 2 | 3 years old | Caregiver struggles with CM (MCS2) | CM-Parent Relationship | 7.94% |
| MCS 2 | 3 years old | parenting: Doing my best for the children | CM-Parent Relationship | 0.00% |
| MCS 2 | 3 years old | parenting: Firm discipline plus lots of fun | CM-Parent Relationship | 0.00% |
| MCS 2 | 3 years old | parenting: Firm rules and discipline | CM-Parent Relationship | 0.00% |
| MCS 2 | 3 years old | parenting: Lots of fun | CM-Parent Relationship | 0.00% |
| MCS 2 | 3 years old | Child-Parent Relationship Scale (CPRS) (MCS2) | CM-Parent Relationship | 6.88% |
| MCS 2 | 3 years old | Conflicts: Child-Parent Relationship Scale (CPRS) (MCS2) | CM-Parent Relationship | 8.92% |
| MCS 2 | 3 years old | Closeness: Child-Parent Relationship Scale (CPRS) (MCS2) | CM-Parent Relationship | 10.00% |
| MCS 2 | 3 years old | Cognition: BAS Naming Vocabulary standardised score (MCS2) | Cognition | 3.90% |
| MCS 2 | 3 years old | Cognition: Bracken School Readiness Composite standardized score (MCS2) | Cognition | 8.15% |
| MCS 2 | 3 years old | Caregiver receives any benefits (MCS2) | Household | 3.08% |
| MCS 2 | 3 years old | Caregiver receives child support (MCS2) | Household | 3.16% |
| MCS 2 | 3 years old | Caregiver has any hobby or leasure activity (MCS2) | Household | 3.07% |
| MCS 2 | 3 years old | Holidays once a year away from home (MCS2) | Household | 3.07% |
| MCS 2 | 3 years old | Satisfaction with home (MCS2) | Household | 3.05% |
| MCS 2 | 3 years old | Satisfaction with area (MCS2) | Household | 3.05% |
| MCS 2 | 3 years old | How safe caregiver feels in the area (MCS2) | Household | 3.05% |
| MCS 2 | 3 years old | Home really disorganised (MCS2) | Household | 3.05% |
| MCS 2 | 3 years old | Calm atmosphere at home (MCS2) | Household | 3.05% |
| MCS 2 | 3 years old | Regular bedtimes (MCS2) | Household | 3.04% |
| MCS 2 | 3 years old | CM eats at regular times (MCS2) | Household | 3.04% |
| MCS 2 | 3 years old | Hours per day spent watching TV or videos (MCS2) | Household | 3.04% |
| MCS 2 | 3 years old | How often caregiver reads to CM (MCS2) | Household | 0.00% |
| MCS 2 | 3 years old | Someone at home help CM to learn sport (MCS2) | Household | 3.04% |
| MCS 2 | 3 years old | Someone at home help CM with alphabet and reading (MCS2) | Household | 3.04% |
| MCS 2 | 3 years old | Someone at home teach CM counting (MCS2) | Household | 3.04% |
| MCS 2 | 3 years old | Someone at home teach CM songs (MCS2) | Household | 3.04% |
| MCS 2 | 3 years old | How often CM paints/draws at home (MCS2) | Household | 4.42% |
| MCS 2 | 3 years old | Family has lots of rules (MCS2) | Household | 3.96% |
| MCS 2 | 3 years old | Rules are stricly enforced (MCS2) | Household | 3.96% |
| MCS 2 | 3 years old | How important for family to eat meals together (MCS2) | Household | 3.96% |
| MCS 2 | 3 years old | Number of carers in household (MCS2) | Household | 0.18% |
| MCS 2 | 3 years old | Number of siblings in household (MCS2) | Household | 0.00% |
| MCS 2 | 3 years old | Half siblings in household (MCS2) | Household | 0.00% |
| MCS 2 | 3 years old | OECD below 60% median poverty indicator (MCS2) | Household | 0.17% |
| MCS 2 | 3 years old | Housing tenure (MCS2) | Household | 0.82% |
| MCS 2 | 3 years old | Caregiver age at birth of CM | Peri and post-natal | 0.00% |
| MCS 3 | 5 years old | Caregiver's general health | Caregiver Health and Mental Health | 0.00% |
| MCS 3 | 5 years old | Caregiver's health limits physical activities | Caregiver Health and Mental Health | 0.29% |
| MCS 3 | 5 years old | Caregiver's health limits work/study | Caregiver Health and Mental Health | 0.83% |
| MCS 3 | 5 years old | Caregiver has longstanding illness | Caregiver Health and Mental Health | 0.02% |
| MCS 3 | 5 years old | How often caregiver drinks alcohol | Caregiver Health and Mental Health | 0.00% |
| MCS 3 | 5 years old | Health/emotional problems limit social activity with family/friends | Caregiver Health and Mental Health | 0.58% |
| MCS 3 | 5 years old | Caregiver used recreational drugs in the past 12 months | Caregiver Health and Mental Health | 2.76% |
| MCS 3 | 5 years old | Caregiver satisfied with partner's work/family balance | Caregiver Health and Mental Health | 20.44% |
| MCS 3 | 5 years old | Caregiver satisfied with own life so far | Caregiver Health and Mental Health | 2.31% |
| MCS 3 | 5 years old | Caregiver Kessler (K6) Scale | Caregiver Health and Mental Health | 1.64% |
| MCS 3 | 5 years old | Rel_Cohabiting | Caregiver Relations | 0.00% |
| MCS 3 | 5 years old | Rel_Married | Caregiver Relations | 0.00% |
| MCS 3 | 5 years old | Rel_Neither | Caregiver Relations | 0.00% |
| MCS 3 | 5 years old | Caregiver marital status | Caregiver Relations | 0.03% |
| MCS 3 | 5 years old | How happy caregiver is with relationship with partner | Caregiver Relations | 14.63% |
| MCS 3 | 5 years old | How often caregiver and partner disagree regarding CM's issues | Caregiver Relations | 17.00% |
| MCS 3 | 5 years old | Partner ever used force in relationship | Caregiver Relations | 16.22% |
| MCS 3 | 5 years old | Natural mother's BMI at interview | Caregiver Relations | 14.84% |
| MCS 3 | 5 years old | Relationship between parents/carers in household | Caregiver Relations | 12.85% |
| MCS 3 | 5 years old | Days per week CM does sport/exercise | Child Health | 3.63% |
| MCS 3 | 5 years old | CM's sleeping habits a problem | Child Health | 3.64% |
| MCS 3 | 5 years old | CM eats at regular times | Child Health | 3.63% |
| MCS 3 | 5 years old | Child's general level of health | Child Health | 3.64% |
| MCS 3 | 5 years old | CM taking regular medication | Child Health | 3.66% |
| MCS 3 | 5 years old | CM has longstanding illness | Child Health | 3.67% |
| MCS 3 | 5 years old | Days per week CM eats breakfast | Child Health | 3.63% |
| MCS 3 | 5 years old | Portions of fruit per day | Child Health | 3.64% |
| MCS 3 | 5 years old | Concern for CM becoming overweight | Child Health | 3.64% |
| MCS 3 | 5 years old | CM enjoys school | Child Mental Health | 4.81% |
| MCS 3 | 5 years old | How often CM reluctant to go to school | Child Mental Health | 4.83% |
| MCS 3 | 5 years old | CM diagnosed with autism/Asperger's | Child Mental Health | 3.63% |
| MCS 3 | 5 years old | CM diagnosed with ADHD | Child Mental Health | 3.69% |
| MCS 3 | 5 years old | How often CM wets bed at night | Child Mental Health | 3.81% |
| MCS 3 | 5 years old | Time spent with friends outside school | Child Relations | 3.63% |
| MCS 3 | 5 years old | How close parent is to CM | CM-Parent Relationship | 1.68% |
| MCS 3 | 5 years old | Parenting competence | CM-Parent Relationship | 2.12% |
| MCS 3 | 5 years old | cognitive score: naming | Cognition | 0.00% |
| MCS 3 | 5 years old | cognitive score: similarity | Cognition | 0.20% |
| MCS 3 | 5 years old | cognitive score: pattern | Cognition | 0.23% |
| MCS 3 | 5 years old | gender | Gender | 0.00% |
| MCS 3 | 5 years old | IMD 2004 Overall Decile_y | Household | 0.02% |
| MCS 3 | 5 years old | Gets help with reading | Household | 4.86% |
| MCS 3 | 5 years old | Gets help with writing | Household | 4.84% |
| MCS 3 | 5 years old | Gets help with maths | Household | 4.84% |
| MCS 3 | 5 years old | Hours per term-time weekday watching tv/dvd | Household | 3.66% |
| MCS 3 | 5 years old | Satisfaction with education at current school | Household | 1.50% |
| MCS 3 | 5 years old | CM has midday meal provided by school | Household | 4.81% |
| MCS 3 | 5 years old | Friends live in the area | Household | 3.64% |
| MCS 3 | 5 years old | Friends and family live in the area | Household | 3.63% |
| MCS 3 | 5 years old | Home is really disorganized | Household | 3.66% |
| MCS 3 | 5 years old | Cannot hear yourself think at home | Household | 3.69% |
| MCS 3 | 5 years old | Atmosphere is calm at home | Household | 3.67% |
| MCS 3 | 5 years old | Good area for raising children | Household | 3.69% |
| MCS 3 | 5 years old | How safe they feel in the area | Household | 3.64% |
| MCS 3 | 5 years old | Own outright, mortgage/loan, part rent/part mortgage | Household | 3.93% |
| MCS 3 | 5 years old | NVQ equivalent of highest qualification across sweeps 1,2 and 3 | Household | 0.09% |
| MCS 3 | 5 years old | Number of siblings in household | Household | 0.00% |
| MCS 3 | 5 years old | OECD Score (derived) | Household | 0.06% |
| MCS 3 | 5 years old | OECD equivalised income (derived) | Household | 0.00% |
| MCS 3 | 5 years old | OECD below 60% median poverty indicator | Household | 0.06% |
| MCS 3 | 5 years old | Number of parents/carers in household | Household | 0.00% |
| MCS 4 | 7 years old | Natural mother BMI (MCS4) | Caregiver Health and Mental Health | 14.66% |
| MCS 4 | 7 years old | Caregiver Kessler (K6) Scale (MCS4) | Caregiver Health and Mental Health | 1.61% |
| MCS 4 | 7 years old | Caregiver Neuroticism subscale (OCEAN) (MCS4) | Caregiver Health and Mental Health | 4.98% |
| MCS 4 | 7 years old | Caregiver Extravert subscale (OCEAN) (MCS4) | Caregiver Health and Mental Health | 7.63% |
| MCS 4 | 7 years old | Frequency caregiver consumes alcohol (MCS4) | Caregiver Health and Mental Health | 0.00% |
| MCS 4 | 7 years old | Satisfaction with work/family balance (MCS4) | Caregiver Health and Mental Health | 29.69% |
| MCS 4 | 7 years old | Satisfaction with job (MCS4) | Caregiver Health and Mental Health | 29.67% |
| MCS 4 | 7 years old | Caregiver experienced racism (MCS4) | Caregiver Health and Mental Health | 5.83% |
| MCS 4 | 7 years old | BMI (MCS4) | Child Health | 0.71% |
| MCS 4 | 7 years old | Anyone smokes near CM (MCS4) | Child Health | 3.58% |
| MCS 4 | 7 years old | Days per week CM does sport/exercise (MCS4) | Child Health | 3.57% |
| MCS 4 | 7 years old | CM general level of health (MCS4) | Child Health | 3.41% |
| MCS 4 | 7 years old | CM taking regular medication (MCS4) | Child Health | 3.58% |
| MCS 4 | 7 years old | Days per week CM eats breakfast (MCS4) | Child Health | 3.48% |
| MCS 4 | 7 years old | CM has a variety of food (MCS4) | Child Health | 3.41% |
| MCS 4 | 7 years old | CM: how often feels happy (MCS4) | Child Mental Health | 5.39% |
| MCS 4 | 7 years old | CM: how often worried (MCS4) | Child Mental Health | 5.71% |
| MCS 4 | 7 years old | CM: how often feels sad (MCS4) | Child Mental Health | 5.60% |
| MCS 4 | 7 years old | CM: how often is quiet (MCS4) | Child Mental Health | 5.75% |
| MCS 4 | 7 years old | CM: how often likes to be alone (MCS4) | Child Mental Health | 5.52% |
| MCS 4 | 7 years old | CM: how often laughs (MCS4) | Child Mental Health | 5.42% |
| MCS 4 | 7 years old | CM: how often lose temper (MCS4) | Child Mental Health | 5.75% |
| MCS 4 | 7 years old | CM: how well get along with siblings (MCS4) | Child Mental Health | 14.77% |
| MCS 4 | 7 years old | CM: how often have fun with family on weekends (MCS4) | Child Mental Health | 5.71% |
| MCS 4 | 7 years old | CM: how much likes school (MCS4) | Child Mental Health | 5.83% |
| MCS 4 | 7 years old | CM: feel safe in the playground (MCS4) | Child Mental Health | 6.13% |
| MCS 4 | 7 years old | CM: how often feels unhappy at school (MCS4) | Child Mental Health | 5.96% |
| MCS 4 | 7 years old | CM: get tired at school (MCS4) | Child Mental Health | 5.84% |
| MCS 4 | 7 years old | CM: get fed up at school (MCS4) | Child Mental Health | 4.93% |
| MCS 4 | 7 years old | CM enjoys school (MCS4) | Child Mental Health | 3.72% |
| MCS 4 | 7 years old | CM reluctant to go to school (MCS4) | Child Mental Health | 3.72% |
| MCS 4 | 7 years old | CM bullied at school (MCS4) | Child Mental Health | 3.90% |
| MCS 4 | 7 years old | Difficulties with maths (MCS4) | Child Mental Health | 3.84% |
| MCS 4 | 7 years old | Difficulties with reading (MCS4) | Child Mental Health | 3.73% |
| MCS 4 | 7 years old | Difficulties with writing (MCS4) | Child Mental Health | 3.78% |
| MCS 4 | 7 years old | Difficulties wih physical education (MCS4) | Child Mental Health | 3.81% |
| MCS 4 | 7 years old | CM receives help with reading (MCS4) | Child Mental Health | 3.72% |
| MCS 4 | 7 years old | CM receives help with writing (MCS4) | Child Mental Health | 3.73% |
| MCS 4 | 7 years old | CM receives help with maths (MCS4) | Child Mental Health | 3.73% |
| MCS 4 | 7 years old | How often CM wets bed at night (MCS4) | Child Mental Health | 3.41% |
| MCS 4 | 7 years old | CM diagnosed with ADHD (MCS4) | Child Mental Health | 3.52% |
| MCS 4 | 7 years old | CM diagnosed with ASD (MCS4) | Child Mental Health | 3.49% |
| MCS 4 | 7 years old | CM: number of friends (MCS4) | Child Relations | 4.95% |
| MCS 4 | 7 years old | CM has best friends (MCS4) | Child Relations | 5.28% |
| MCS 4 | 7 years old | CM enjoys playing with friends (MCS4) | Child Relations | 4.90% |
| MCS 4 | 7 years old | CM: how often bullied by other children (MCS4) | Child Relations | 5.34% |
| MCS 4 | 7 years old | CM: how often feel left out (MCS4) | Child Relations | 5.07% |
| MCS 4 | 7 years old | How close caregiver is to CM (MCS4) | CM-Parent Relationship | 1.61% |
| MCS 4 | 7 years old | How often caregiver expresses affection by hugging or kissing (MCS4) | CM-Parent Relationship | 1.56% |
| MCS 4 | 7 years old | How often caregiver gets irritated with CM (MCS4) | CM-Parent Relationship | 1.59% |
| MCS 4 | 7 years old | How often caregiver enjoys listening and doing things with CM (MCS4) | CM-Parent Relationship | 1.56% |
| MCS 4 | 7 years old | Cognition: Word reading scores standardised (MCS4) | Cognition | 2.32% |
| MCS 4 | 7 years old | Cognition: Number skills (MCS4) | Cognition | 0.82% |
| MCS 4 | 7 years old | Cognition: Pattern Construction standardised (MCS4) | Cognition | 0.99% |
| MCS 4 | 7 years old | Maths scores standardised (MCS4) | Cognition | 0.82% |
| MCS 4 | 7 years old | Number of carers in household (MCS4) | Household | 0.00% |
| MCS 4 | 7 years old | Number of siblings in household (MCS4) | Household | 0.02% |
| MCS 4 | 7 years old | Half siblings in household (MCS4) | Household | 0.00% |
| MCS 4 | 7 years old | Step siblings in household (MCS4) | Household | 0.00% |
| MCS 4 | 7 years old | Adoptive siblings in household (MCS4) | Household | 0.00% |
| MCS 4 | 7 years old | Housing tenure (MCS4) | Household | 0.41% |
| MCS 4 | 7 years old | OECD below 60% median poverty indicator (MCS4) | Household | 0.02% |
| MCS 4 | 7 years old | Whether caregiver works mainly from home (MCS4) | Household | 29.44% |
| MCS 4 | 7 years old | Home really disorganised (MCS4) | Household | 3.64% |
| MCS 4 | 7 years old | Friends and family live in the area (MCS4) | Household | 3.61% |
| MCS 4 | 7 years old | Clubs or classes outside school (MCS4) | Household | 3.57% |
| MCS 4 | 7 years old | Hours per weekday spend watching TV or videos (MCS4) | Household | 3.58% |
| MCS 4 | 7 years old | Hours per weekday playing on computer (MCS4) | Household | 3.64% |
| MCS 4 | 7 years old | How often caregiver reads to CM (MCS4) | Household | 0.02% |
| MCS 4 | 7 years old | Midday meal provided by school (MCS4) | Household | 8.33% |
| MCS 5 | 11 years old | Caregiver general health (MCS5) | Caregiver Health and Mental Health | 0.17% |
| MCS 5 | 11 years old | Caregiver longstanding illness | Caregiver Health and Mental Health | 0.18% |
| MCS 5 | 11 years old | Caregiver smokes (MCS5) | Caregiver Health and Mental Health | 0.17% |
| MCS 5 | 11 years old | How often caregiver plays sport/physical activity (MCS5) | Caregiver Health and Mental Health | 0.17% |
| MCS 5 | 11 years old | How often caregiver felt depressed in last 30 days (MCS5) | Caregiver Health and Mental Health | 2.09% |
| MCS 5 | 11 years old | How often caregiver felt hopeless in last 30 days (MCS5) | Caregiver Health and Mental Health | 1.97% |
| MCS 5 | 11 years old | How often caregiver felt restless in last 30 days (MCS5) | Caregiver Health and Mental Health | 1.84% |
| MCS 5 | 11 years old | How often caregiver felt worthless in last 30 days (MCS5) | Caregiver Health and Mental Health | 2.05% |
| MCS 5 | 11 years old | How often caregiver felt nervous in last 30 days (MCS5) | Caregiver Health and Mental Health | 1.76% |
| MCS 5 | 11 years old | Frequency caregiver consumes alcohol (MCS5) | Caregiver Health and Mental Health | 0.41% |
| MCS 5 | 11 years old | Life satisfaction (MCS5) | Caregiver Health and Mental Health | 2.50% |
| MCS 5 | 11 years old | Happy with relationship with partner (MCS5) | Caregiver Relations | 19.68% |
| MCS 5 | 11 years old | Partner ever used force (MCS5) | Caregiver Relations | 19.82% |
| MCS 5 | 11 years old | Days per week CM does sport/exercise (MCS5) | Child Health | 3.85% |
| MCS 5 | 11 years old | CM has longstanding illness (MCS5) | Child Health | 3.92% |
| MCS 5 | 11 years old | CM general level of health (MCS5) | Child Health | 3.87% |
| MCS 5 | 11 years old | Number of accidents or injuries (MCS5) | Child Health | 3.93% |
| MCS 5 | 11 years old | CM taking regular medication (MCS5) | Child Health | 3.89% |
| MCS 5 | 11 years old | Days per week CM eats breakfast (MCS5) | Child Health | 3.95% |
| MCS 5 | 11 years old | CM has a variety of food (MCS5) | Child Health | 3.89% |
| MCS 5 | 11 years old | BMI (MCS5) | Child Health | 2.12% |
| MCS 5 | 11 years old | CM enjoys school (MCS5) | Child Mental Health | 4.02% |
| MCS 5 | 11 years old | How often CM bored at school (MCS5) | Child Mental Health | 4.04% |
| MCS 5 | 11 years old | Individual support in class from teacher or assistant (MCS5) | Child Mental Health | 4.01% |
| MCS 5 | 11 years old | CM has special classes (MCS5) | Child Mental Health | 4.01% |
| MCS 5 | 11 years old | CM attends a special school (MCS5) | Child Mental Health | 4.01% |
| MCS 5 | 11 years old | How often CM wets bed at night (MCS5) | Child Mental Health | 3.85% |
| MCS 5 | 11 years old | CM diagnosed with ADHD (MCS5) | Child Mental Health | 4.66% |
| MCS 5 | 11 years old | CM diagnosed with ASD (MCS5) | Child Mental Health | 4.64% |
| MCS 5 | 11 years old | CM: feel about school work (MCS5) | Child Mental Health | 1.71% |
| MCS 5 | 11 years old | CM: feel about the school you go to (MCS5) | Child Mental Health | 1.88% |
| MCS 5 | 11 years old | CM: feel about the way you look (MCS5) | Child Mental Health | 2.02% |
| MCS 5 | 11 years old | CM: feel about your family (MCS5) | Child Mental Health | 1.94% |
| MCS 5 | 11 years old | CM: feel about your friends (MCS5) | Child Mental Health | 2.03% |
| MCS 5 | 11 years old | CM: get tired at school (MCS5) | Child Mental Health | 2.40% |
| MCS 5 | 11 years old | CM: how often feel happy (MCS5) | Child Mental Health | 2.50% |
| MCS 5 | 11 years old | CM: how often feel sad (MCS5) | Child Mental Health | 3.20% |
| MCS 5 | 11 years old | CM: how often feel scared (MCS5) | Child Mental Health | 3.23% |
| MCS 5 | 11 years old | CM: how often feel worried (MCS5) | Child Mental Health | 2.81% |
| MCS 5 | 11 years old | CM: how often got angry (MCS5) | Child Mental Health | 3.25% |
| MCS 5 | 11 years old | CM: how often laughed (MCS5) | Child Mental Health | 3.10% |
| MCS 5 | 11 years old | CM: like school (MCS5) | Child Mental Health | 2.32% |
| MCS 5 | 11 years old | CM: on the whole, satisfied with self (MCS5) | Child Mental Health | 3.54% |
| MCS 5 | 11 years old | CM: school is a waste of time (MCS5) | Child Mental Health | 2.40% |
| MCS 5 | 11 years old | How often CM spends time with friends outside school (MCS5) | Child Relations | 3.85% |
| MCS 5 | 11 years old | Allowed unsupervised time outside home with friends (MCS5) | Child Relations | 3.85% |
| MCS 5 | 11 years old | CM: How often do other CM picked on or hurt by on purpose? (MCS5) | Child Relations | 2.19% |
| MCS 5 | 11 years old | CM: How often do siblings picked on or hurt by on purpose? (MCS5) | Child Relations | 9.83% |
| MCS 5 | 11 years old | Amount of time with child (MCS5) | CM-Parent Relationship | 0.05% |
| MCS 5 | 11 years old | CGT: Risk taking (MCS5) | Cognition | 4.39% |
| MCS 5 | 11 years old | Cognition: Verbal similarities standardised (MCS5) | Cognition | 1.14% |
| MCS 5 | 11 years old | How many times holidays outside UK (MCS5) | Household | 3.87% |
| MCS 5 | 11 years old | How often CM visits library (MCS5) | Household | 3.89% |
| MCS 5 | 11 years old | How often CM attends religious service (MCS5) | Household | 3.87% |
| MCS 5 | 11 years old | CM plays a musical instrument | Household | 3.85% |
| MCS 5 | 11 years old | Hours per weekday spend watching TV or videos (MCS5) | Household | 3.85% |
| MCS 5 | 11 years old | Hours per weekday spent on computer or games (MCS5) | Household | 3.90% |
| MCS 5 | 11 years old | CM has computer of their own (MCS5) | Household | 5.83% |
| MCS 5 | 11 years old | CM uses Internet at home (MCS5) | Household | 6.15% |
| MCS 5 | 11 years old | CM has access to Internet on phones (MCS5) | Household | 3.85% |
| MCS 5 | 11 years old | Regular bedtimes (MCS5) | Household | 3.87% |
| MCS 5 | 11 years old | How often CM helps look after disabled family members (MCS5) | Household | 3.87% |
| MCS 5 | 11 years old | Midday meal provided by school (MCS5) | Household | 4.02% |
| MCS 5 | 11 years old | CM has own bedroom (MCS5) | Household | 3.70% |
| MCS 5 | 11 years old | Quiet area where CM can do homework (MCS5) | Household | 3.70% |
| MCS 5 | 11 years old | Any parks where CM can play outdoors (MCS5) | Household | 3.70% |
| MCS 5 | 11 years old | Whether caregiver works mainly from home (MCS5) | Household | 24.57% |
| MCS 5 | 11 years old | Hours per week working (MCS5) | Household | 24.72% |
| MCS 5 | 11 years old | Cannot hear yourself think at home (MCS5) | Household | 3.89% |
| MCS 5 | 11 years old | Good area to bring up child (MCS5) | Household | 3.70% |
| MCS 5 | 11 years old | Friends or family in the area (MCS5) | Household | 3.70% |
| MCS 5 | 11 years old | Caregiver experienced racism (MCS5) | Household | 4.75% |
| MCS 5 | 11 years old | Activity_Employed | Household | 0.00% |
| MCS 5 | 11 years old | Activity_Looking after the family | Household | 0.00% |
| MCS 5 | 11 years old | Activity_Looking for work | Household | 0.00% |
| MCS 5 | 11 years old | Activity_Other | Household | 0.00% |
| MCS 5 | 11 years old | Activity_Self employed | Household | 0.00% |
| MCS 5 | 11 years old | Adoptive siblings in household (MCS5) | Household | 0.00% |
| MCS 5 | 11 years old | CM: How often listen to or play music (MCS5) | Household | 1.79% |
| MCS 5 | 11 years old | CM: How often draw, paint or make things (MCS5) | Household | 1.73% |
| MCS 5 | 11 years old | CM: How often exchange messages with friends on the internet (MCS5) | Household | 2.08% |
| MCS 5 | 11 years old | CM: How often play games on a computer or games console (MCS5) | Household | 1.81% |
| MCS 5 | 11 years old | CM: How often play sports or active games inside or outside (MCS5) | Household | 2.03% |
| MCS 5 | 11 years old | CM: How often read for enjoyment (MCS5) | Household | 1.70% |
| MCS 5 | 11 years old | CM: How often use the internet (MCS5) | Household | 1.78% |
| MCS 5 | 11 years old | CM: How often visit a social networking website on the internet, such as Facebook (MCS5) | Household | 1.99% |
| MCS 5 | 11 years old | CM: wish my family could afford to buy me more of what I want (MCS5) | Household | 2.75% |
| MCS 5 | 11 years old | Caregiver in work (MCS5) | Household | 0.00% |
| MCS 5 | 11 years old | Half siblings in household (MCS5) | Household | 0.00% |
| MCS 5 | 11 years old | Housing tenure (MCS5) | Household | 0.30% |
| MCS 5 | 11 years old | IMD 2004 Overall Decile | Household | 0.06% |
| MCS 5 | 11 years old | NVQ highest academic level across sweeps (MCS5) | Household | 0.00% |
| MCS 5 | 11 years old | Number of carers in household (MCS5) | Household | 0.00% |
| MCS 5 | 11 years old | Number of siblings in household (MCS5) | Household | 0.00% |
| MCS 5 | 11 years old | OECD below 60% median poverty indicator (MCS5) | Household | 0.00% |
| MCS 5 | 11 years old | Step siblings in household (MCS5) | Household | 0.00% |
|  |  |  |  |  |

**Edge cases analyses**

To verify that the proportion of children transitioning to another cluster was not driven by their distance to the cluster centre, we calculated the distribution of the silhouette scores for the individuals who were found to be part of a ‘significant’ transition and compared it to the distribution of silhouette scores for the whole cluster the ‘transitioning’ individuals belonged to. We then performed a two-sample Kolmogorov-Smirnov (KS) test to compare the two distributions. After transforming the data to normal distributions using quantiles information (sklearn.preprocessing.QuantileTransformer), we also performed a two-sample t-test to compare the means of the two distributions. No significant differences were found for all ‘significant transitions’ we tested, which confirm that the transition of individuals from one cluster to another was not confounded by the location of the individuals in the cluster.

**Supplementary Figure 4**

*Transitions from 5 to 11 years-old.*

**
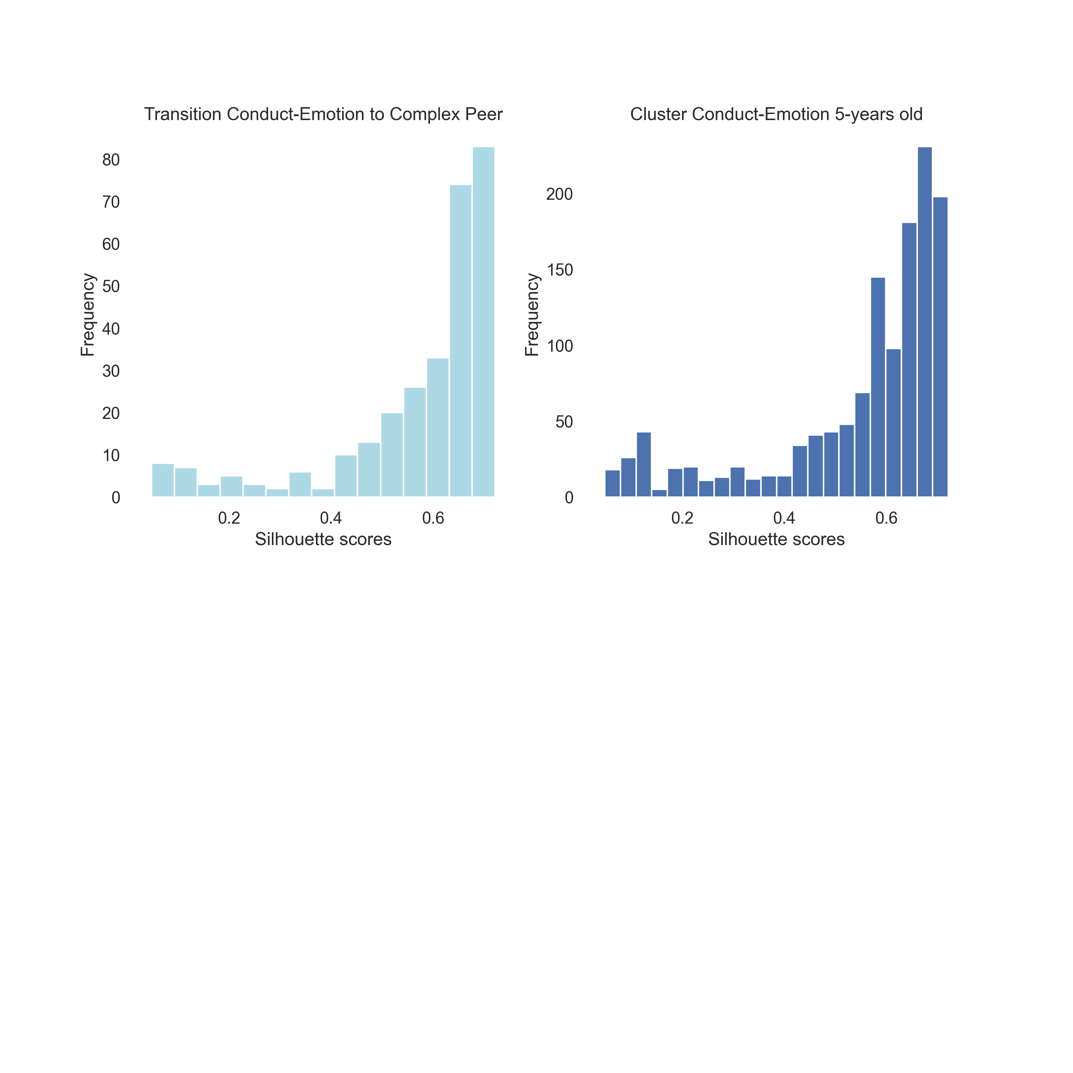

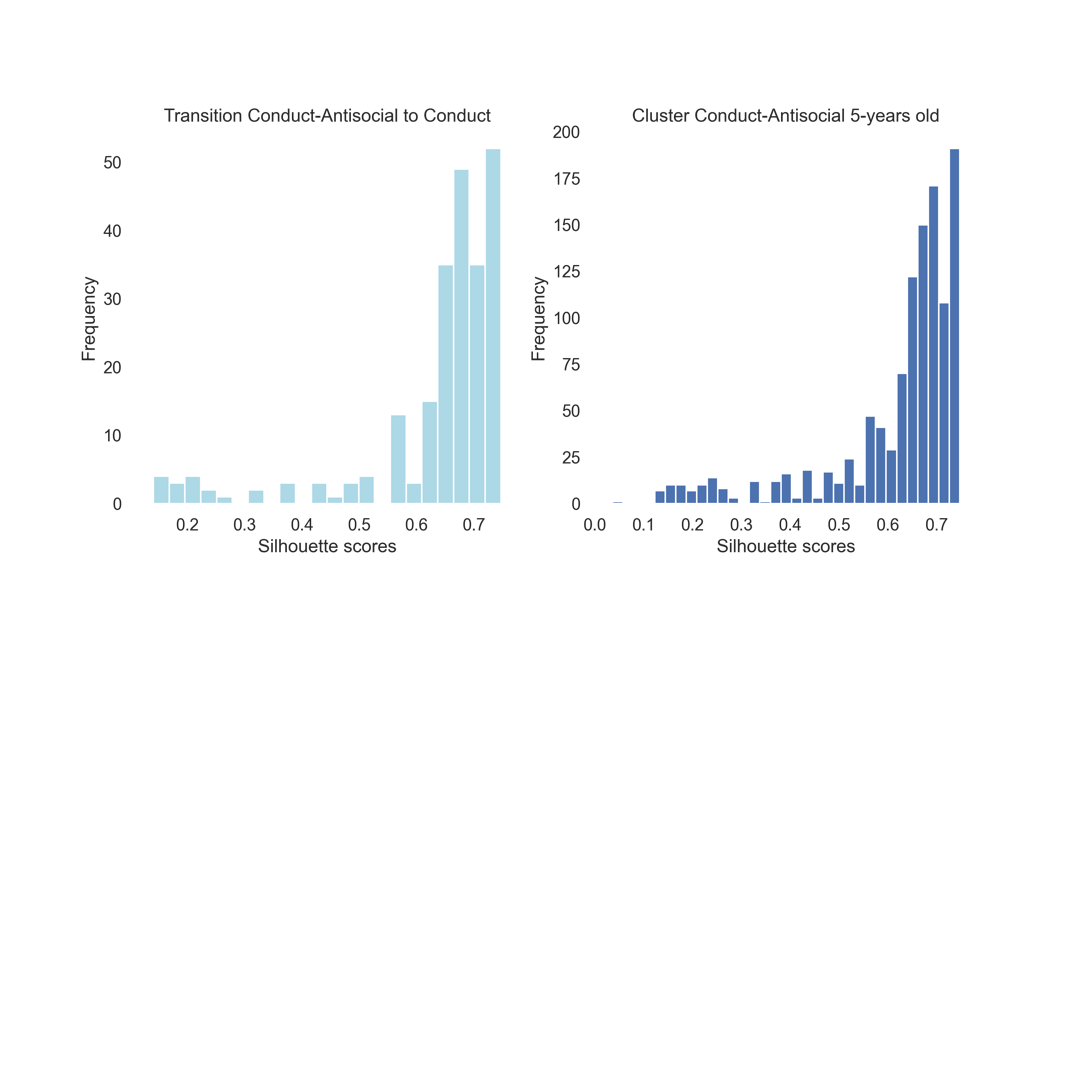
**

*KS test to compare distributions:*

D = 0.073, *p* = 0.24

*t-test to compare means on transformed normal distributions:*

t = -0.001, *p* = 0.99

*KS test to compare distributions:*

D = 0.089, *p* = 0.04

*t-test to compare means on transformed normal distributions:*

t = 0.001, *p* = 0.99

*Note;* Distributions of silhouette scores for ‘significant transitions’ samples (light blue) compared to the whole cluster (dark blue) between ages 5 and 11 years-old.

**Supplementary Figure 5**

*Transitions from 11 to 17 years-old.*

**
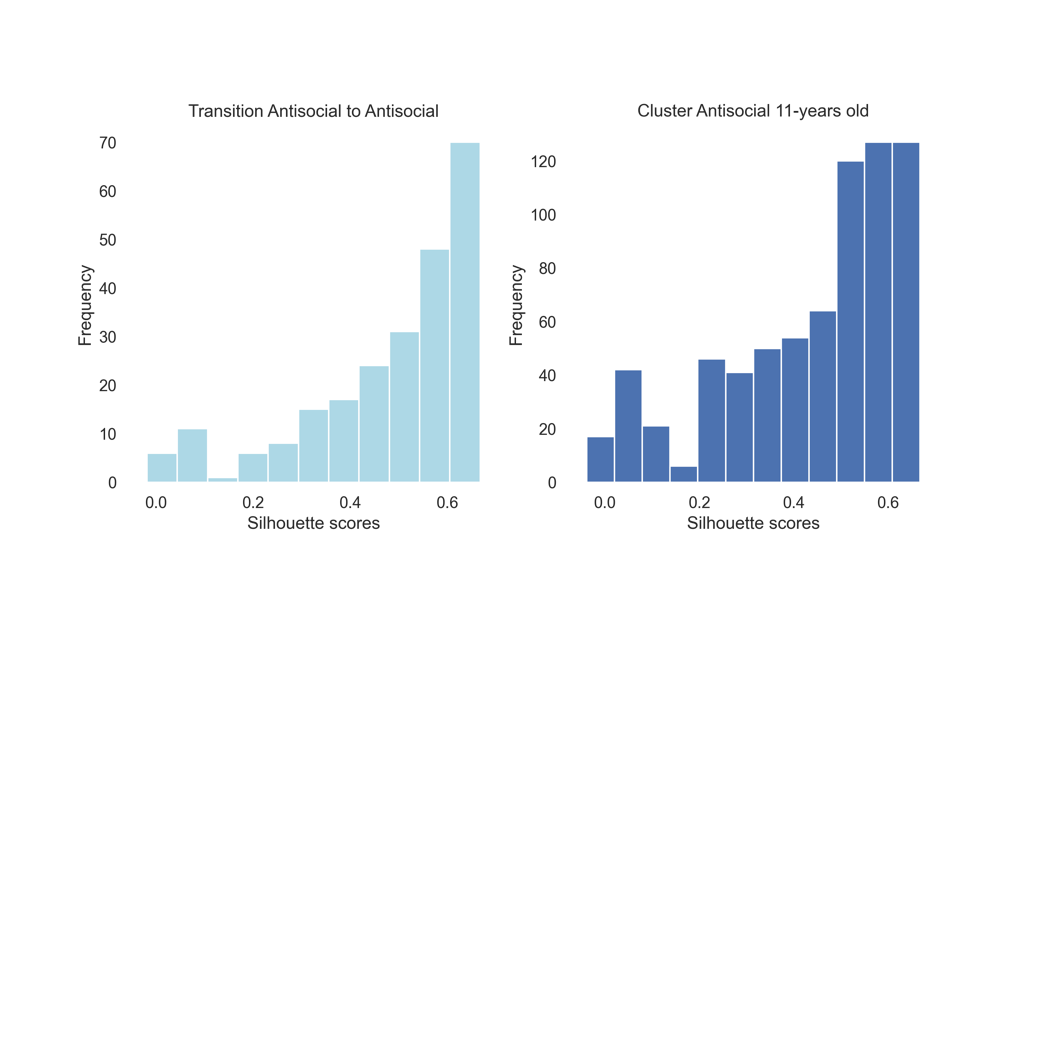

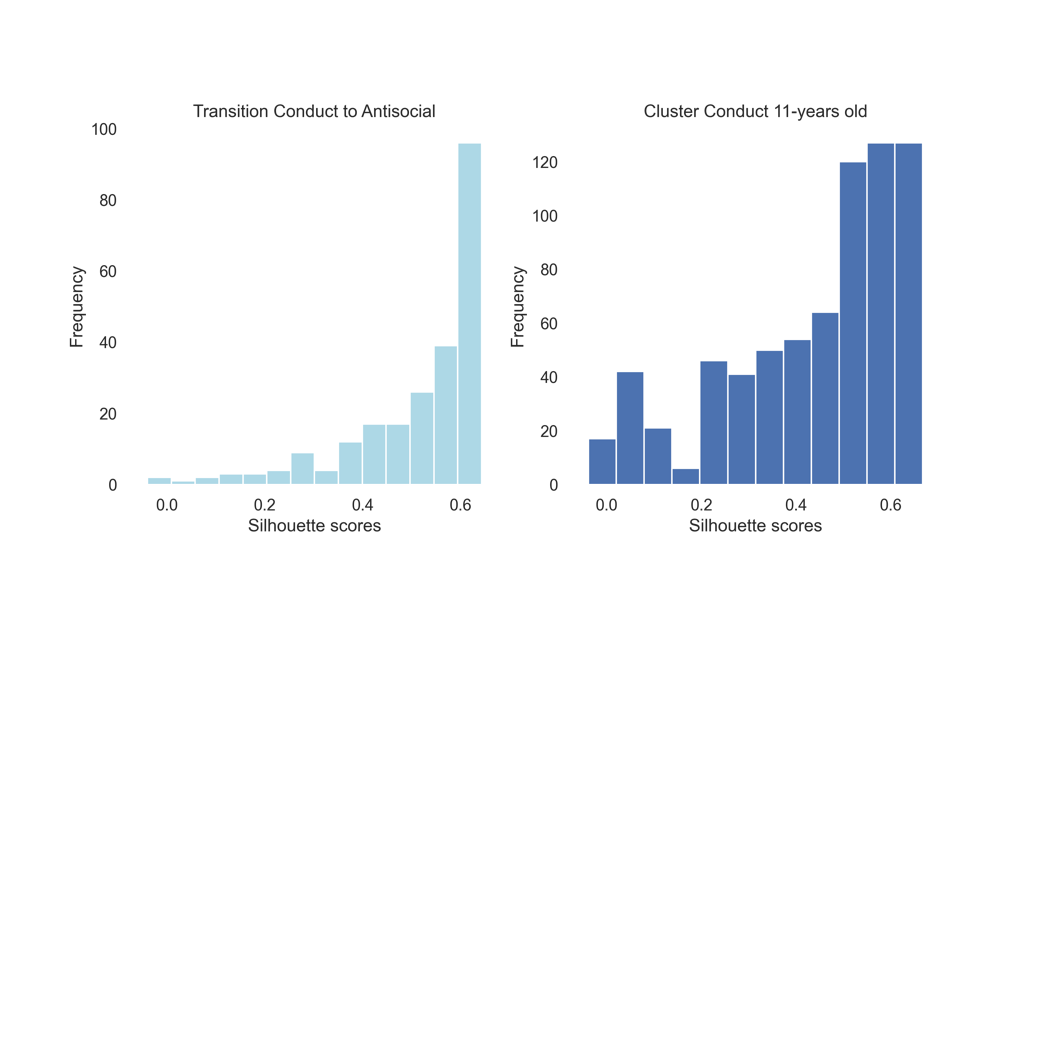

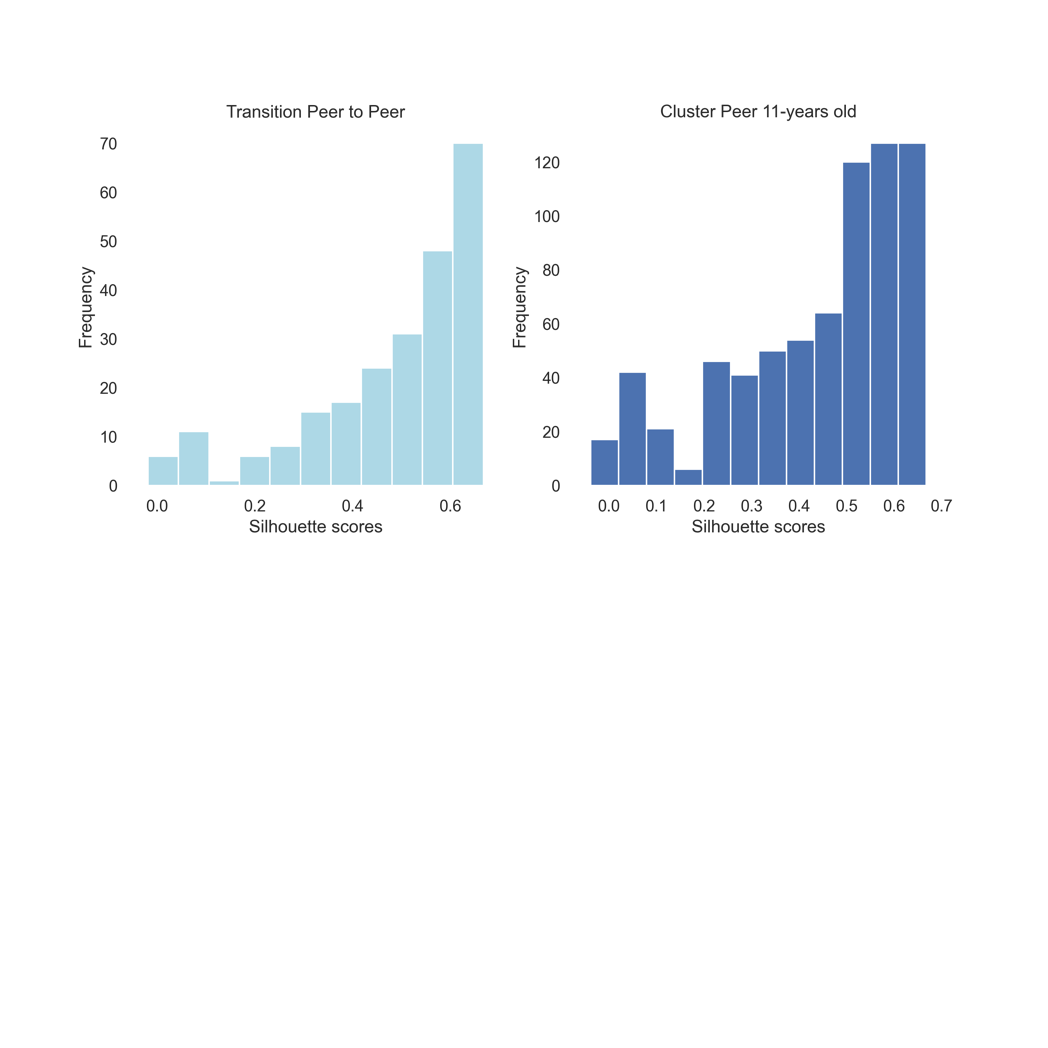
**

*KS test to compare distributions:*

D = 0.12, *p* = 0.01

*t-test to compare means on transformed normal distributions:*

t = 0.001, *p* = 1.0

*KS test to compare distributions:*

D = 0.12, *p* = 0.01

*t-test to compare means on transformed normal distributions:*

t = 0.001, *p* = 1.0

*KS test to compare distributions:*

D = 0.12, *p* = 0.01

*t-test to compare means on transformed normal distributions:*

t < 0.001, *p* = 1.0

*Note;* Distributions of silhouette scores for ‘significant transitions’ samples (light blue) compared to the whole cluster (dark blue) between ages 11 and 17 years-old.
